# A 209‐Site DNA Methylation Predictor of Chronological Age Evaluated Under Empirical and Random Missingness

**DOI:** 10.1111/acel.70800

**Published:** 2026-11-01

**Authors:** Xiangteng Zhao, Yanhong Lu, Biqing Huang, Zijie Wang, Tong Yu, Ruoqi Zhao, Shiwen Yu, Shushan Wei, Yeting Zeng

**Affiliations:** ^1^ Department of Neurology Zhongshan Hospital Affiliated to Fudan University Shanghai China; ^2^ Department of Emergency The First Affiliated Hospital of Fujian Medical University Fuzhou China; ^3^ Department of Nuclear Medicine Shengli Clinical Medical College of Fujian Medical University, Fujian Provincial Hospital, Fuzhou University Affiliated Provincial Hospital Fuzhou China; ^4^ Fujian Research Institute of Nuclear Medicine Fuzhou China; ^5^ Department of Neurosurgery Fujian Provincial Hospital, Fuzhou University Affiliated Provincial Hospital Fuzhou China; ^6^ Department of Neurology Shengli Clinical Medical College of Fujian Medical University, Fujian Provincial Hospital, Fuzhou University Affiliated Provincial Hospital Fuzhou China

**Keywords:** chronological age, DNA methylation, formalin‐fixed paraffin‐embedded, missing‐data sensitivity, nearest‐neighbor reconstruction, technical missingness

## Abstract

DNA methylation age prediction from incomplete profiles requires evaluation under empirical and random missingness. We evaluated whole‐blood chronological‐age prediction using 524 development and 132 held‐out GSE40279 samples and an external GSE73103 cohort (*n* = 355). Feature‐stability analysis across 750 prespecified perturbation conditions identified a 209‐CpG primary panel and a 52‐CpG stringent sensitivity subset. The primary predictor combined five‐nearest‐neighbor reconstruction (KNN5) with Ridge regression. In the internal holdout, intact‐profile prediction achieved a mean absolute error of 4.24 years, a root‐mean‐square error (RMSE) of 5.38 years, and Pearson *r* = 0.934. Under masks derived from 42 empirical templates of formalin‐fixed paraffin‐embedded detection failure, KNN5 reduced RMSE by 0.365 years relative to development‐set mean replacement (95% bootstrap interval for the difference, −0.546 to −0.205), with larger reductions at higher missingness burdens. The reconstruction advantage persisted in complementary development analyses after randomization preserving both sample‐level and CpG‐level failure counts. External predictions remained associated with chronological age (*r* = 0.835), despite age‐dependent calibration bias. Mixture analyses showed that background accuracy was central to target recovery as mixture asymmetry increased. These findings support local reconstruction within a stability‐selected CpG panel for chronological‐age prediction under the evaluated empirical and random missingness conditions.

## Graphical abstract

A stability‐selected 209‐CpG panel with nearest‐neighbor reconstruction supports chronological‐age prediction from incomplete blood methylation profiles. In held‐out samples under empirically derived missingness masks, reconstruction reduced root‐mean‐square error by 0.365 years versus mean replacement, with larger benefits at higher missingness burdens.

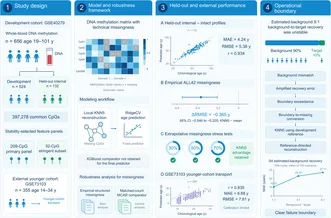

## 1 Introduction

DNA methylation varies systematically with chronological age and can support quantitative age estimation in blood and other tissues. Seminal blood‐based and multi‐tissue clocks established the feasibility of predicting chronological age from CpG measurements (Hannum et al. 2013; Horvath 2013), whereas subsequent work distinguished chronological‐age prediction from epigenetic measures intended to capture broader aspects of biological aging (Bell et al. 2019; Field et al. 2018; Horvath and Raj 2018). Later generations of epigenetic aging measures, including DNAm PhenoAge, GrimAge, and pace‐of‐aging metrics, were developed to capture aging‐related phenotypes, mortality risk, or the rate of biological aging rather than chronological age per se (Belsky et al. 2020; Levine et al. 2018; Lu et al. 2019; Oblak et al. 2021). The present study therefore focuses exclusively on chronological age. Forensic studies have further supported compact blood‐based age‐prediction panels, particularly those containing ELOVL2, across targeted and sequencing‐based assays (Garagnani et al. 2012; Naue et al. 2017; Park et al. 2016; Vidaki et al. 2017; Weidner et al. 2014; Zbieć‐Piekarska et al. 2015a, 2015b), including inter‐laboratory implementation (Garali et al. 2020).

A practical limitation is that archived, low‐input, fragmented, or formalin‐fixed paraffin‐embedded (FFPE) specimens can exhibit sample‐ and probe‐dependent measurement failure, whereas methylation age predictors are typically developed and evaluated using profiles in which the required predictor CpGs are available. DNA damage and degraded‐template artifacts are established analytical concerns (Alaeddini et al. 2010; Do and Dobrovic 2015; Hanssen et al. 2017; Llamas et al. 2012), and low‐input methylation profiling introduces additional technical constraints (Naue et al. 2018). FFPE array studies further demonstrate specimen‐ and probe‐dependent variation in restoration and detection performance (de Ruijter et al. 2015; Kling et al. 2017; Siegel et al. 2014). We derived empirical templates from detection‐P failures in GSE38266, an independent FFPE HumanMethylation450 head‐and‐neck squamous cell carcinoma dataset (Lechner et al. 2013). They retain observed failed‐probe identities and joint occurrence, complementing burden‐matched random masks. Only failure patterns, not tumor methylation values, were transferred; these are assay‐failure templates rather than a universal biochemical model of DNA degradation.

Handling missing measurements can influence both feature selection and subsequent prediction. Mean or median replacement assigns missing CpGs a population‐level central value, whereas nearest‐neighbor reconstruction estimates missing values from reference samples that remain similar across the observed features (Dong and Peng 2013; Sterne et al. 2009; Troyanskaya et al. 2001; Waljee et al. 2013). Other nonparametric approaches similarly demonstrate that imputation performance depends on the structure of the observed data (Stekhoven and Bühlmann 2012). Feature stability presents a related challenge because methylation predictors are high‐dimensional and correlated, and perturbations in missingness, data partitioning, or preprocessing can yield overlapping but nonidentical selected feature sets. Elastic net is well suited to correlated predictors (Zou and Hastie 2005). Downstream model class can also affect prediction independently of feature selection and reconstruction, including when nonlinear approaches such as XGBoost are considered (Chen and Guestrin 2016). These considerations motivated separate analyses of selected‐feature stability, fold‐contained reconstruction, and downstream regression.

Mixture recovery presents a related but distinct identifiability problem. For a mixture containing a target fraction α, linear subtraction scales measurement noise by 1/α and background‐estimation error by (1‐α)/α, making both sources of error increasingly consequential as the target fraction decreases. Methylation and cell‐free‐DNA deconvolution studies illustrate why source composition generally has to be estimated rather than assumed to be known (Houseman et al. 2012; Moss et al. 2018; Snyder et al. 2016). More broadly, reliable evaluation of a prediction model requires separation of model development from test data and joint consideration of prediction error, association, systematic bias, and calibration‐related behavior (Collins et al. 2015; Stevens and Poppe 2020; Van Calster et al. 2019).

To address these issues, we separated model development from held‐out internal evaluation and external transportability assessment. Model development used 524 GSE40279 samples, while empirically observed FFPE‐associated detection failures derived from an independent dataset provided templates of structured technical missingness. Feature stability was evaluated across missingness seeds, cross‐validation partitions, elastic‐net mixing parameters, and preprocessing strategies, while reconstruction and downstream regression were assessed as separate components of the prediction pipeline. The predictor and the prespecified primary internal endpoint were fixed before evaluation in the remaining 132 GSE40279 samples. GSE73103, comprising 355 healthy young individuals (Voisin et al. 2015), was subsequently used to assess external transportability without refitting or recalibration. Complementary feature‐perturbation and mixture‐recovery analyses further characterized predictive dependence on individual features and the limits of recovery under background uncertainty. Within this framework, we quantified the reduction in chronological‐age prediction error obtained with local reconstruction under empirical and random missingness and evaluated how prediction performance was influenced by missingness burden, between‐cohort differences, feature‐set instability, and background‐estimation error.

## 2 Results

### 2.1 Multi‐Axis Stability Analysis Defines a 209‐CpG Primary Panel for Held‐Out Evaluation

To identify CpGs that remained stable across perturbations of the development procedure, we evaluated feature membership across 750 prespecified conditions in the 524‐sample GSE40279 development set and compared reconstruction and regression strategies. These analyses defined the 209‐CpG KNN5‐RidgeCV predictor. The remaining GSE40279 samples and GSE73103 were subsequently evaluated without feature reselection, model refitting, or recalibration (Figure 1A).

**FIGURE 1:**
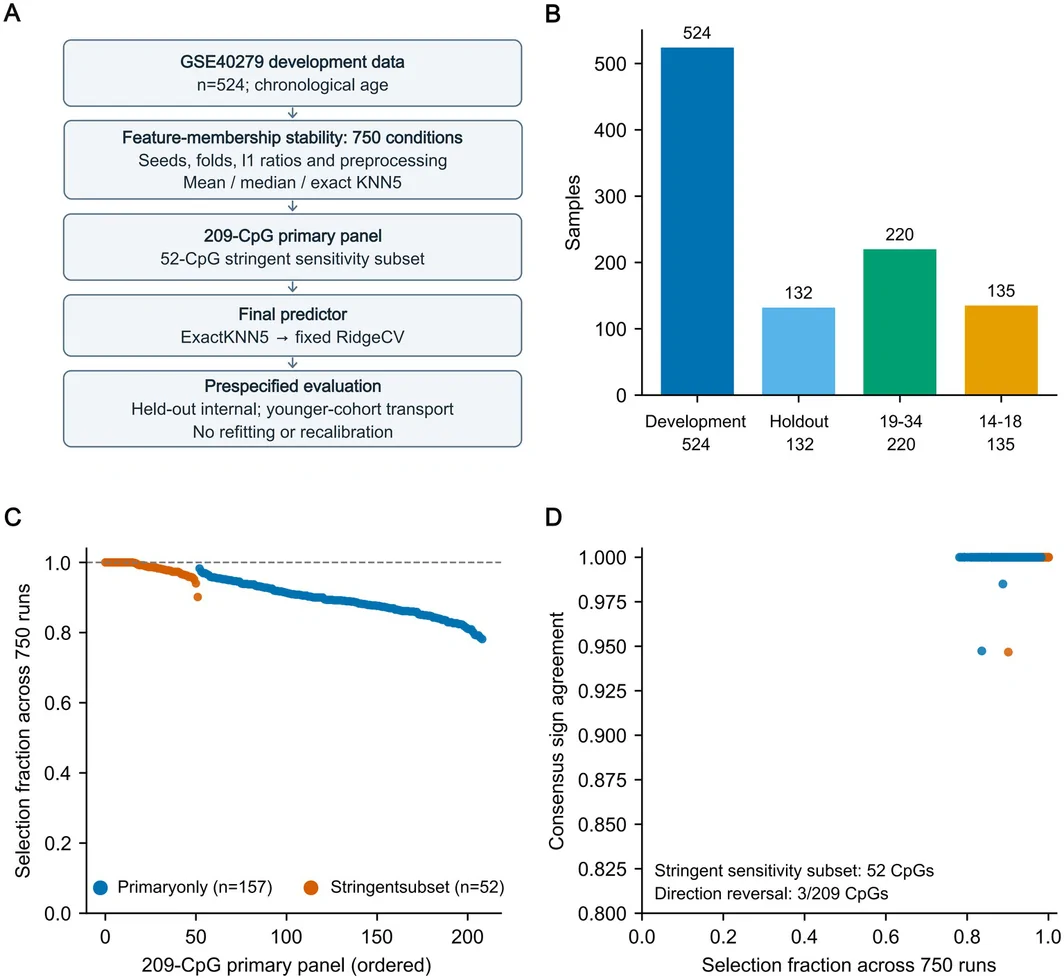
Study design and selection stability of the 209‐CpG age‐prediction panel. (A) Development of the 209‐CpG chronological‐age predictor in the GSE40279 development set, followed by held‐out internal evaluation and younger‐cohort transport without feature reselection, model refitting, or recalibration. (B) Cohort composition and sample sizes for GSE40279 development (*n* = 524), GSE40279 held‐out internal evaluation (*n* = 132), and external transport in GSE73103 participants aged 19–34 years (*n* = 220) and 14–18 years (*n* = 135). (C) Selection fractions of the 209 CpGs across 750 prespecified development conditions spanning missingness seeds, cross‐validation partitions, elastic‐net mixing parameters, and preprocessing strategies. Blue points denote the 157 CpGs retained only in the primary panel; orange points denote the 52‐CpG stringent sensitivity subset. (D) Relationship between selection fraction and consensus coefficient‐sign agreement across the same conditions. CpG, cytosine‐phosphate‐guanine site; KNN5, five‐nearest‐neighbor reconstruction.

GSE40279 comprised 656 whole‐blood methylation profiles and was divided by age‐decile‐by‐sex stratification into 524 development samples and 132 held‐out internal samples. GSE73103 was concentrated at younger ages and was therefore retained as an external transport cohort rather than pooled with GSE40279; its 355 samples included 220 participants aged 19–34 years and 135 aged 14–18 years. The ordered CpG intersection between the two cohorts contained 397,278 sites. Chronological age was the sole modeled outcome, and neither disease status nor biological‐age phenotypes entered model development or evaluation (Figure 1B).

We next examined whether CpG membership remained stable when missingness seeds, elastic‐net mixing parameters, cross‐validation partitions, and preprocessing strategies were varied jointly. Across the 750 development conditions, the 209‐CpG primary panel had a median selection fraction of 0.908 (interquartile range, 0.871–0.959; mean, 0.913), and every CpG was selected in at least 78.1% of conditions (Figure 1C). Fifty‐two CpGs satisfied the more stringent criterion across all evaluated l1‐ratio settings and were retained as a sensitivity subset; their position within the ordered selection‐frequency distribution is shown in Supplementary Figure 1A.

Coefficient direction was similarly consistent across the perturbation grid. Median consensus‐sign agreement was 1.000, the minimum was 0.947, and only three of the 209 CpGs showed at least one coefficient‐sign reversal (Figure 1D). Median paired Jaccard similarity between corresponding 3‐fold and 5‐fold feature sets was 1.000, whereas increasing the elastic‐net l1 ratio produced progressively more selective feature retention (Supplementary Figure 1B). cg16867657 (ELOVL2), a well‐established age‐associated CpG, was included in the 52‐CpG stringent subset. Together, these results support the use of the 209‐CpG panel as the primary panel for held‐out evaluation, with the 52‐CpG subset providing a more stringent sensitivity benchmark.

### 2.2 Increasing Structured Missingness Reduces Feature Stability Across Empirical and Randomized Failure Patterns

To model structured technical missingness using observed assay failures, we derived 42 templates from independently measured FFPE‐associated detection failures in GSE38266. The templates were grouped as S1 (< 1%), S2 (1%–< 5%), S3 (5%–< 20%), and S4 (≥ 20%) according to missingness burden, with ALL42 denoting the complete set. Empirical masks preserved the observed failed‐probe identities and within‐template co‐failure structure, column‐permuted masks preserved burden and recurrence while disrupting probe identity, and matched‐count MCAR masks preserved each sample's missing‐CpG count while randomizing the affected sites. Across the 397,278 common CpGs, mean empirical missingness was 0.261% for S1, 6.245% for ALL42, and 29.351% for S4 (Figure 2A). Projection of the same template families onto the 209‐CpG panel in the internal holdout produced mean missingness burdens of 0.620%, 9.022%, and 37.879%, respectively, across ten mask assignments.

**FIGURE 2:**
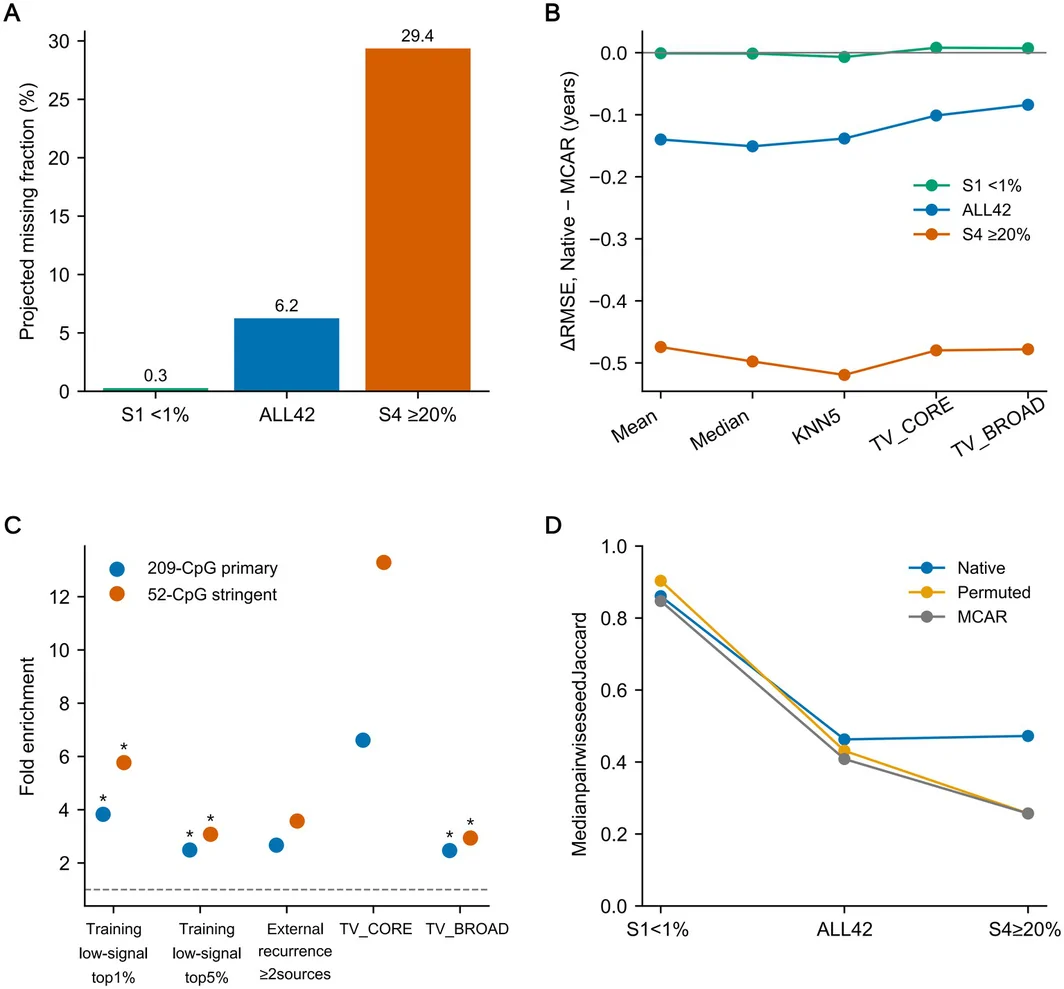
Empirical and random missingness, technical susceptibility, and feature stability. (A) Mean missing fraction across the 397,278 common CpGs under S1, ALL42, and S4 empirical (Native) FFPE‐derived masks. (B) Difference in root‐mean‐square error (RMSE) between empirical (Native) and matched‐count MCAR masks (Native minus MCAR) in development‐set elastic‐net analyses across mean replacement, median replacement, KNN5 reconstruction, and technical‐vulnerability exclusion settings. Negative values indicate lower RMSE under Native masks. (C) Enrichment of the 209‐CpG primary panel and 52‐CpG stringent sensitivity subset in independently defined technical‐susceptibility annotations. Asterisks denote Benjamini‐Hochberg‐adjusted *q* < 0.05 from one‐sided hypergeometric tests across 12 comparisons. (D) Median pairwise Jaccard similarity of selected CpG sets across ten mask‐assignment seeds under empirical (Native), column‐permuted, and matched‐count MCAR masks. All analyses were performed in the 524‐sample GSE40279 development set. S1, < 1% missingness; S4, ≥ 20% missingness; ALL42, all 42 FFPE‐derived templates. Native, empirical probe‐failure pattern; TV_CORE and TV_BROAD, core and broad technical‐vulnerability sets excluded in the corresponding sensitivity analyses; FFPE, formalin‐fixed paraffin‐embedded; KNN5, five‐nearest‐neighbor reconstruction; MCAR, missing completely at random.

We compared Native and matched‐count MCAR masks using condition‐specific elastic‐net models fitted in the development set. With development‐set mean replacement, empirical‐minus‐MCAR RMSE differences were −0.140 years for ALL42 (BH *q* = 0.0308) and −0.474 years for S4 (*q* = 0.00879) (Figure 2B). The same direction was observed across preprocessing and technical‐susceptibility sensitivity analyses, with ALL42 differences of approximately −0.084 to −0.151 years and S4 differences of −0.474 to −0.519 years (Supplementary Figure 2A). Empirical‐versus‐column‐permuted contrasts were smaller and less consistent. Negative Native‐minus‐MCAR differences indicate lower RMSE under Native masks in these comparisons, not greater damage from the empirical patterns.

We next examined whether the age‐prediction panels were enriched for independently defined markers of technical susceptibility. The 209‐CpG panel was enriched 3.83‐fold among the training‐derived lowest‐signal 1% of probes (global BH *q* = 0.00534) and 2.49‐fold among the lowest‐signal 5% (*q* = 0.000118). Corresponding enrichments for the 52‐CpG subset were 5.77‐fold (*q* = 0.0307) and 3.08‐fold (*q* = 0.0123) (Figure 2C). The broad vulnerability set (TV_BROAD; 20,804 CpGs) was enriched in both panels, whereas TV_CORE (575 CpGs) and the most recurrent external‐failure set were not globally significant. These enrichments support an association between the selected age‐prediction CpGs and technical susceptibility, but they do not isolate a causal effect because excluding a vulnerability set also changes both feature availability and projected missingness.

Finally, feature‐selection stability declined as missingness burden increased. Median pairwise Jaccard similarities for empirical, column‐permuted, and matched‐count MCAR masks were 0.861, 0.903, and 0.847 under S1, respectively, but decreased to 0.463, 0.431, and 0.408 under ALL42 (Figure 2D). Under S4, median similarity was 0.472 for empirical masks and approximately 0.257 for both randomized control structures. A similar burden‐dependent decline was observed across preprocessing and vulnerability‐exclusion analyses (Supplementary Figure 2B). Together, these results show that increasing missingness burden was consistently associated with lower selected‐feature stability across empirical and randomized failure structures.

### 2.3 KNN5 Reconstruction Lowers Prediction Error Under Structured Missingness, With Effects Modified by Feature Representation

Having defined the primary panel, we separated the contributions of reconstruction and downstream regression. In intact profiles, XGBoost produced an RMSE 0.506 years higher than Ridge with either mean or KNN5 preprocessing, as no values required reconstruction (Figure 3A). When missingness was introduced, KNN5 reduced RMSE relative to training‐mean replacement with Ridge by 0.231–0.333 years across the three ALL42 mask variants and by 1.294–1.596 years across the three S4 variants (Figure 3B). After KNN5 reconstruction, XGBoost remained 0.205–0.505 years higher in RMSE than Ridge across the nine masked conditions (Figure 3C). The 52‐CpG stringent subset did not consistently reduce RMSE relative to the 209‐CpG primary panel (Figure 3D; Supplementary Figure 3A). These fixed‐panel comparisons supported the 209‐CpG KNN5‐RidgeCV specification used for held‐out evaluation.

**FIGURE 3:**
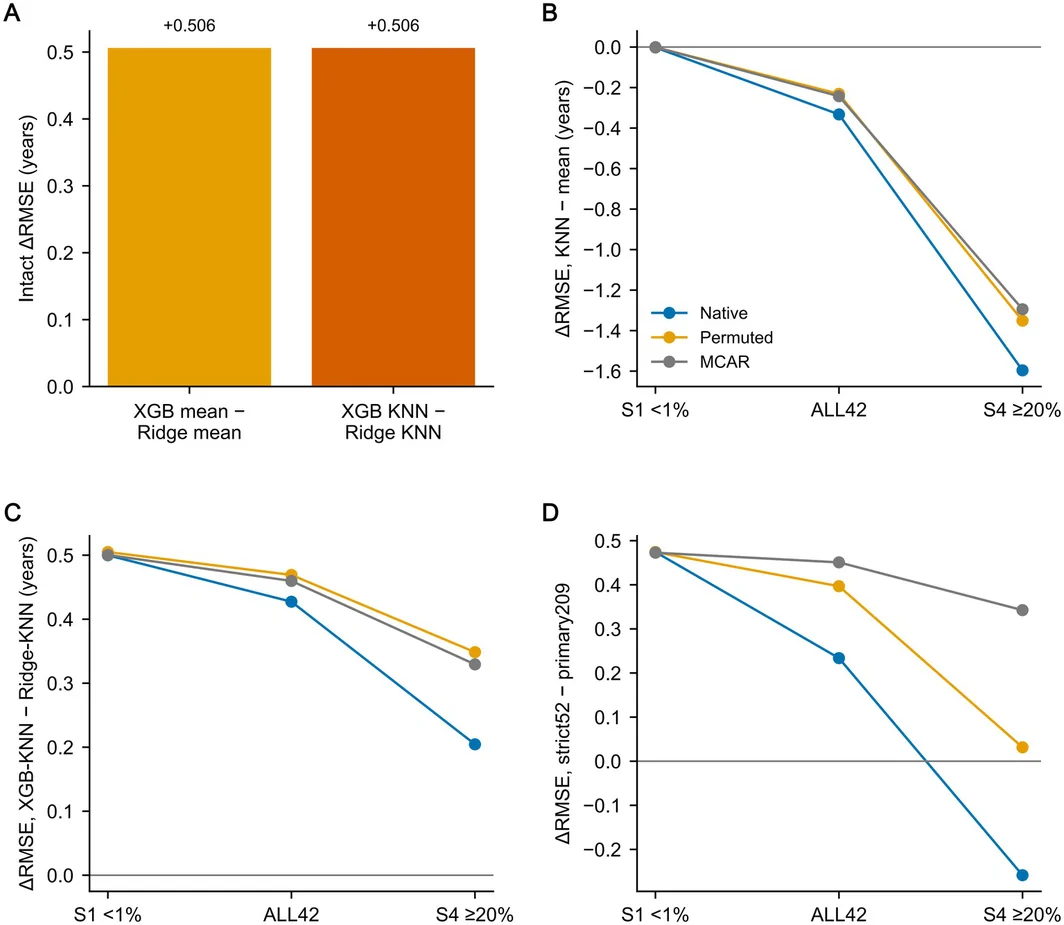
Performance comparisons of reconstruction, regression, and CpG‐panel size. (A) Difference in intact‐profile root‐mean‐square error (RMSE) between XGBoost and Ridge regression under development‐set mean replacement and KNN5 preprocessing, respectively (XGBoost minus Ridge). (B) Difference in RMSE between KNN5 reconstruction and development‐set mean replacement with Ridge regression (KNN5 minus mean). (C) Difference in RMSE between XGBoost and Ridge regression after KNN5 reconstruction (XGBoost minus Ridge). (D) Difference in RMSE between the 52‐CpG stringent sensitivity subset and the 209‐CpG primary panel using Ridge regression and development‐set mean replacement (52‐CpG minus 209‐CpG). For masked analyses, estimates were averaged across ten mask assignments under S1, ALL42, and S4 empirical (Native), column‐permuted, and matched‐count MCAR masks. S1, < 1% missingness; S4, ≥ 20% missingness; ALL42, all 42 FFPE‐derived templates; FFPE, formalin‐fixed paraffin‐embedded; KNN5, five‐nearest‐neighbor reconstruction; MCAR, missing completely at random.

An independent development‐only 2 × 2 × 2 factorial analysis evaluated feature space, reconstruction, and regression without altering the held‐out predictor. Expanded elastic‐net regularization yielded interior relative α values and retained 3093–3243 CpGs across folds. Under ALL42 Native masks, MAE/RMSE ranged from 5.335/7.249 years for all‐CpG mean‐Ridge to 4.345/5.710 years for selected KNN5‐XGBoost; absolute MAE changes from the original regularization ranges were ≤ 0.011 years (Supplementary Figure 4A; Table 1; Supplementary Table 1). Ridge reached the expanded lower bound in the all‐CpG and fold‐selected EN representations. In a separate fixed‐209 sensitivity analysis, all five outer folds selected interior positive αs (1.0 or 2.154), with OOF MAE/RMSE of 3.503/4.810 years versus 4.364/5.725 years for OLS. Ridge boundary behavior was therefore feature‐representation dependent (Supplementary Table 1).

**TABLE 1:** Reconstruction and regression comparisons under ALL42 empirical (Native) masks with expanded regularization ranges.

Feature space	Reconstruction	Regressor	MAE (years)	RMSE (years)	MAE change (years)
All CpGs	Mean	Ridge	5.335	7.249	−0.000003
All CpGs	Mean	XGBoost	4.858	6.361	+0.000000
All CpGs	KNN5	Ridge	5.227	7.161	−0.000003
All CpGs	KNN5	XGBoost	4.808	6.299	+0.000000
Fold‐selected EN	Mean	Ridge	5.168	6.904	+0.005978
Fold‐selected EN	Mean	XGBoost	4.482	5.921	−0.010309
Fold‐selected EN	KNN5	Ridge	4.882	6.619	+0.011035
Fold‐selected EN	KNN5	XGBoost	4.345	5.710	−0.002317

*Note:* Errors are averaged across ten mask assignments in the 524‐sample GSE40279 development set evaluated by five‐fold outer cross‐validation. “All CpGs” denotes the 397,278 common CpGs; fold‐specific elastic‐net models retained 3093–3243 CpGs. MAE change was calculated as expanded‐grid minus original‐grid MAE.

Abbreviations: EN, elastic net; KNN5, five‐nearest‐neighbor reconstruction; MAE, mean absolute error; RMSE, root‐mean‐square error.

Averaged across the other factorial terms, feature selection, KNN5 reconstruction, and XGBoost changed MAE by −0.338, −0.145, and −0.530 years, with Bonferroni‐adjusted percentile intervals of [−0.481, −0.191], [−0.216, −0.074], and [−0.964, −0.121], respectively (Supplementary Figure 4B). The selection‐by‐KNN5 interaction was negative, whereas the KNN5‐by‐XGBoost and three‐way interactions were positive; the selection‐by‐XGBoost interval included zero. The regressor contrast also depended on feature representation: with KNN5, XGBoost‐minus‐Ridge MAE was −0.537 years for fold‐selected features but +0.414 years for the separately evaluated fixed 209‐CpG panel (Supplementary Figure 4C). Because this factorial selector differed from the 750‐condition stability procedure, these component contrasts were interpreted as context‐dependent effects rather than as a ranking of fully optimized algorithms.

We further tested the fixed 209‐CpG panel across three constructed missingness mechanisms. Under co‐failure‐module masks, KNN5 versus mean‐replacement MAE was 4.002 versus 5.108 years at 30% loss, 4.491 versus 6.651 years at 50%, and 5.619 versus 8.719 years at 70% (Supplementary Figure 5A); the corresponding RMSE series is shown in Supplementary Figure 5B. The same burden sequence was then evaluated with susceptibility‐weighted masks (Supplementary Figure 5C,D) and exact‐count MCAR masks (Supplementary Figure 5E,F). Across all three mechanisms, absolute MAE and RMSE increased as missingness increased, while KNN5 remained lower than mean replacement. The paired KNN5‐minus‐mean contrasts are summarized for MAE and RMSE in Supplementary Figure 5G,H, and all 18 individual conditional intervals excluded zero (Supplementary Table 2). Because marginal failure frequencies differed across mechanisms and the co‐failure modules were only partially sampled, these comparisons do not isolate dependence alone and should not be interpreted as genomic‐spatial effects.

To control marginal failure burden directly, we randomized each co‐failure mask while preserving every sample and CpG failure count. Under these margin‐preserving randomizations, KNN5 MAE was 4.007, 4.532, and 5.650 years at nominal 30%, 50%, and 70% loss, compared with 5.118, 6.675, and 8.732 years after mean replacement (Table 2; Supplementary Table 3). KNN5‐minus‐mean contrasts remained negative under both mask types, with adjusted intervals excluding zero. By contrast, co‐failure‐minus‐randomized KNN5 MAE differences were −0.005, −0.041, and −0.031 years, and their adjusted intervals included zero; the mask‐by‐reconstruction interactions also included zero. Thus, the lower error associated with KNN5 persisted after matching both row and column failure margins, whereas an additional co‐failure effect was not detected. These conditional comparisons do not establish equivalence between the two mask types.

**TABLE 2:** Reconstruction performance under co‐failure masks and marginal‐count‐preserving randomization.

Nominal loss	Co‐failure KNN5/mean MAE (years)	Randomized KNN5/mean MAE (years)	KNN5 mask difference ΔMAE [adjusted interval]	Interaction ΔMAE [adjusted interval]
30%	4.002/5.108	4.007/5.118	−0.005 [−0.069, 0.057]	+0.005 [−0.048, 0.060]
50%	4.491/6.651	4.532/6.675	−0.041 [−0.116, 0.031]	−0.017 [−0.086, 0.050]
70%	5.619/8.719	5.650/8.732	−0.031 [−0.157, 0.085]	−0.018 [−0.127, 0.085]

*Note:* MAE is reported in years. Values for co‐failure and randomized masks are shown as KNN5 reconstruction/development‐set mean replacement. Nominal 30%, 50%, and 70% loss correspond to 63, 105, and 146 missing CpGs per sample, respectively. Randomization preserves both sample‐level and CpG‐level failure counts. The KNN5 mask difference is defined as co‐failure minus randomized MAE, and the interaction as the co‐failure KNN5‐minus‐mean contrast minus the corresponding randomized contrast. Brackets show Bonferroni‐adjusted percentile intervals across 15 prespecified MAE contrasts based on 5000 paired subject resamples. Estimates were averaged across ten masks and, for randomized masks, four chains per mask. Analyses used the 524‐sample GSE40279 development set and the predefined 209‐CpG panel.

Abbreviations: KNN5, five‐nearest‐neighbor reconstruction; MAE, mean absolute error.

### 2.4 Feature Perturbation Identifies Predictive Concentration With Partial Redundancy

We next examined how predictive information was distributed across the 209‐CpG panel and whether correlated features could compensate when highly ranked CpGs were removed. Within each outer training fold, feature importance was defined as the absolute Ridge coefficient multiplied by the training‐fold feature standard deviation. Prediction‐error change was expressed as ΔRMSE, calculated as RMSE after perturbation minus the corresponding unperturbed RMSE within the same context. Structural deletion removed the top‐ranked 1%, 5%, 10%, 20%, or 40% of the 209‐CpG panel (2, 10, 21, 42, or 84 CpGs). Top‐ranked deletions were compared with the mean ΔRMSE across 200 random deletion sets (Supplementary Figure 6A) and with bottom‐ranked deletions at the same doses (Supplementary Figure 6B). Each structural deletion was followed by re‐imputation and Ridge refitting, allowing the remaining CpGs to compensate.

At 5% structural deletion, targeted‐minus‐random RMSE increases were 0.0032 years for intact profiles (BH *q* = 0.490), 0.030 years under ALL42 Native masks (*q* = 0.218), and 0.1634 years under S4 Native masks (*q* = 0.00933). Increasing the deletion dose amplified the contrast: at 20%, the corresponding increases were 0.268, 0.224, and 0.245 years, and at 40% they were 0.492, 0.421, and 0.294 years (Supplementary Figure 6A). The separation between top‐ranked and bottom‐ranked deletions likewise increased with dose (Supplementary Figure 6B).

A complementary post‐KNN5 value‐permutation analysis assessed reliance on highly ranked CpGs without allowing re‐imputation or model refitting. Reconstructed values for top‐ranked CpGs were permuted within each outer validation fold while the fitted predictor was retained, and the resulting ΔRMSE was compared with the mean across 200 random‐feature permutation sets (Supplementary Figure 6C). At the 5% permutation dose, targeted‐minus‐random RMSE increases were 0.739 years for intact profiles, 0.786 years under ALL42 Native masks, and 0.864 years under S4 Native masks (BH q = 0.004975 for each). cg16867657 (ELOVL2) was consistently highly ranked across the five outer training folds, with ranks of 4, 2, 6, 7, and 2 (Supplementary Figure 6D).

Across the full dose series, structural‐deletion effects increased progressively (Supplementary Figure 7A), whereas post‐KNN5 value permutation produced larger targeted‐minus‐random contrasts at corresponding doses (Supplementary Figure 7B). MAE followed the same overall pattern, with metric‐specific significance. In the MAE analysis, intact profiles were evaluated first by structural deletion and permutation (Supplementary Figure 8A,B), followed in the same paired order by ALL42 Native (C,D), S4 Native (E,F), S1 Native (G,H), ALL42 MCAR (I,J), and S4 MCAR (K,L). At 5% structural deletion, targeted‐minus‐random delta‐MAE was 0.0513, 0.0603, and 0.1385 years for intact, ALL42 Native, and S4 Native profiles (BH *q* = 0.0459, 0.0249, and 0.00829). At 5% post‐KNN5 value permutation, the corresponding contrasts were 0.7892, 0.8008, and 0.8144 years (*q* = 0.004975 for each). Supplementary Table 4 reports all 120 MAE/RMSE comparisons.

Taken together, highly ranked CpGs carried more predictive information than matched random sets in the fitted model, with the largest contrasts observed when reconstructed values were perturbed without refitting. The smaller effects after structural deletion are consistent with partial compensation by correlated CpGs after re‐imputation and model refitting. These analyses therefore characterize predictive concentration and redundancy within the fitted predictor rather than biological causality.

### 2.5 KNN5 Lowers Held‐Out Prediction Error Under Structured Technical Missingness

After model specification was completed using development data, the 132‐sample GSE40279 holdout was evaluated once. On intact profiles, predicted age closely tracked chronological age, with an MAE of 4.240 years, an RMSE of 5.381 years, and a Pearson correlation of 0.934 (Figure 4A). Residuals showed a mean bias of −0.086 years and an age‐on‐prediction slope of 1.073; younger individuals tended to be overpredicted and older individuals underpredicted despite the near‐zero mean bias (Figure 4B).

**FIGURE 4:**
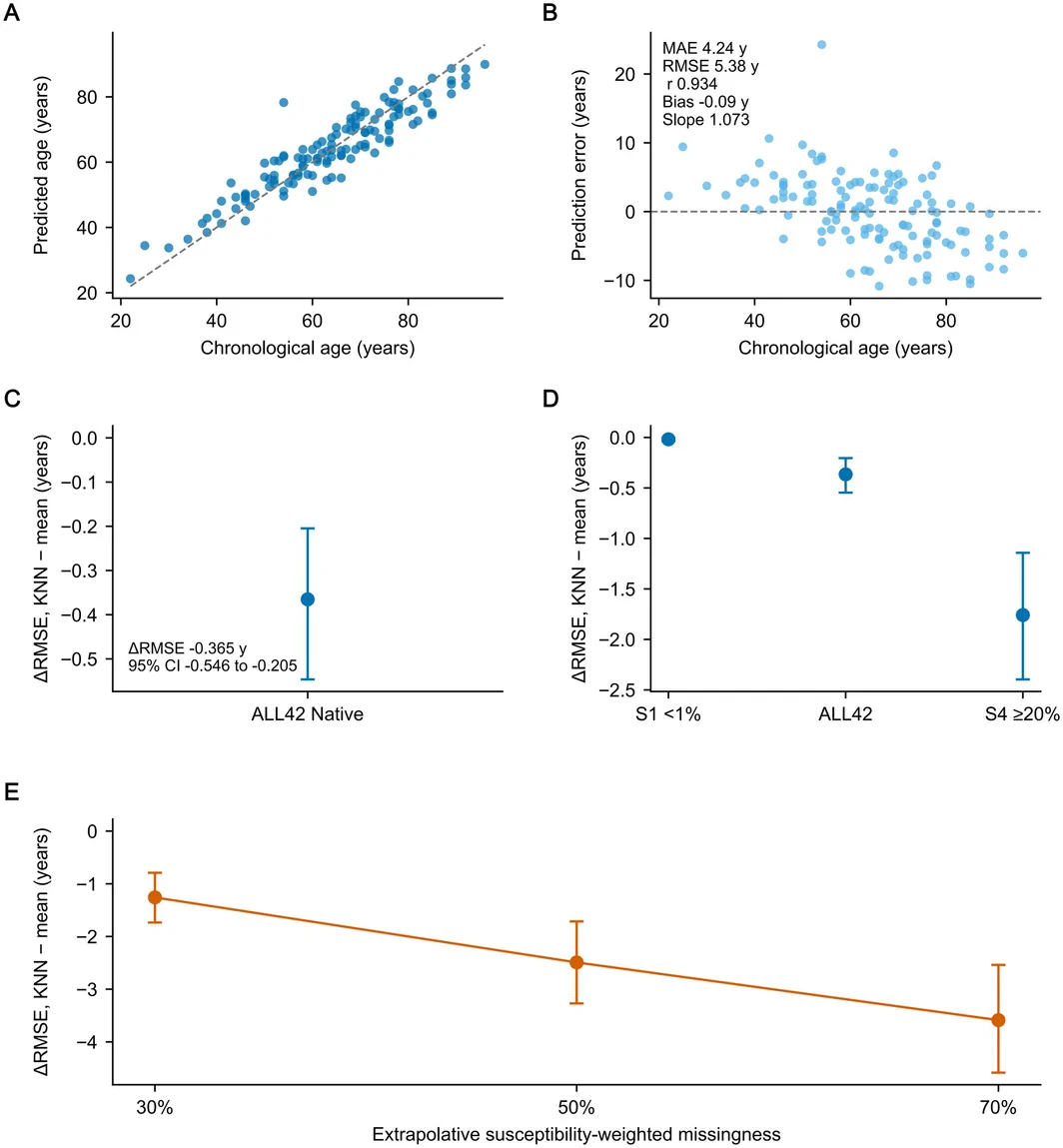
Held‐out internal performance under structured technical missingness. (A) Predicted versus chronological age in the GSE40279 held‐out internal set (*n* = 132) using the 209‐CpG KNN5‐RidgeCV predictor. The dashed line denotes identity. (B) Prediction residuals, calculated as predicted minus chronological age. The dashed line denotes zero residual. (C) Difference in root‐mean‐square error (RMSE) between KNN5 reconstruction and development‐set mean replacement under ALL42 empirical (Native) masks (KNN5 minus mean). (D) The same RMSE contrast under S1, ALL42, and S4 empirical (Native) masks. (E) KNN5‐minus‐mean RMSE differences under susceptibility‐weighted masks with nominal missingness of 30%, 50%, and 70%. For masked analyses, points represent means across ten mask assignments, and error bars denote 95% percentile intervals from 2000 paired subject‐level bootstrap resamples. S1, < 1% missingness; S4, ≥ 20% missingness; ALL42, all 42 FFPE‐derived templates; KNN5, five‐nearest‐neighbor reconstruction; FFPE, formalin‐fixed paraffin‐embedded; RMSE, root‐mean‐square error.

The prespecified primary endpoint compared KNN5 reconstruction with development‐set mean replacement using the same Ridge coefficients under ALL42 Native masks. Averaged across ten mask assignments, RMSE was 0.365 years lower with KNN5 (KNN5‐minus‐mean difference, −0.365 years; 95% sample‐level bootstrap interval, −0.546 to −0.205; 2000 replicates), and every bootstrap contrast was below zero (Figure 4C). The same contrast increased in magnitude across the empirical Native burden series, from −0.018 years under S1 to −0.365 years under ALL42 and −1.759 years under S4 (Figure 4D).

We next extended this comparison to susceptibility‐weighted stress tests with nominal 30%, 50%, and 70% missingness. These fixed‐burden conditions were derived from empirical susceptibility weights and were not treated as observed whole‐blood FFPE distributions. At 30% missingness, KNN5 yielded MAE/RMSE values of 4.589/5.756 years compared with 5.640/7.014 years after development‐mean replacement, corresponding to an RMSE reduction of 1.258 years. At 50%, MAE/RMSE was 5.099/6.377 years with KNN5 and 7.130/8.869 years with mean replacement, a 2.492‐year RMSE reduction. At 70%, the corresponding values were 6.073/7.532 and 8.899/11.119 years, a 3.587‐year RMSE reduction (Figure 4E). Thus, the absolute separation between reconstruction strategies increased with missingness burden, although prediction error also increased for both approaches.

When empirical and constructed conditions were placed on a common missingness axis, Native‐minus‐matched‐count MCAR RMSE differences with KNN5 were −0.042 years for ALL42 and −0.214 years for S4, with intervals including zero (Supplementary Figure 9A). These mask‐structure contrasts were substantially smaller than the KNN5‐versus‐mean reconstruction differences observed at comparable burdens.

### 2.6 External Transport Preserves Age Association but Reveals Age‐Dependent Calibration Bias

We applied the fixed predictor to GSE73103 without refitting or recalibration. Predicted age remained associated with chronological age, but the two prespecified age strata differed visibly: participants aged 14–18 years (*n* = 135) showed greater dispersion and stronger underestimation than those aged 19–34 years (*n* = 220) (Figure 5A). In the full cohort (*n* = 355), MAE was 6.680 years and RMSE was 7.608 years; corresponding values were 5.748 and 6.634 years at ages 19–34 years and 8.198 and 8.971 years at ages 14–18 years (Figure 5B).

**FIGURE 5:**
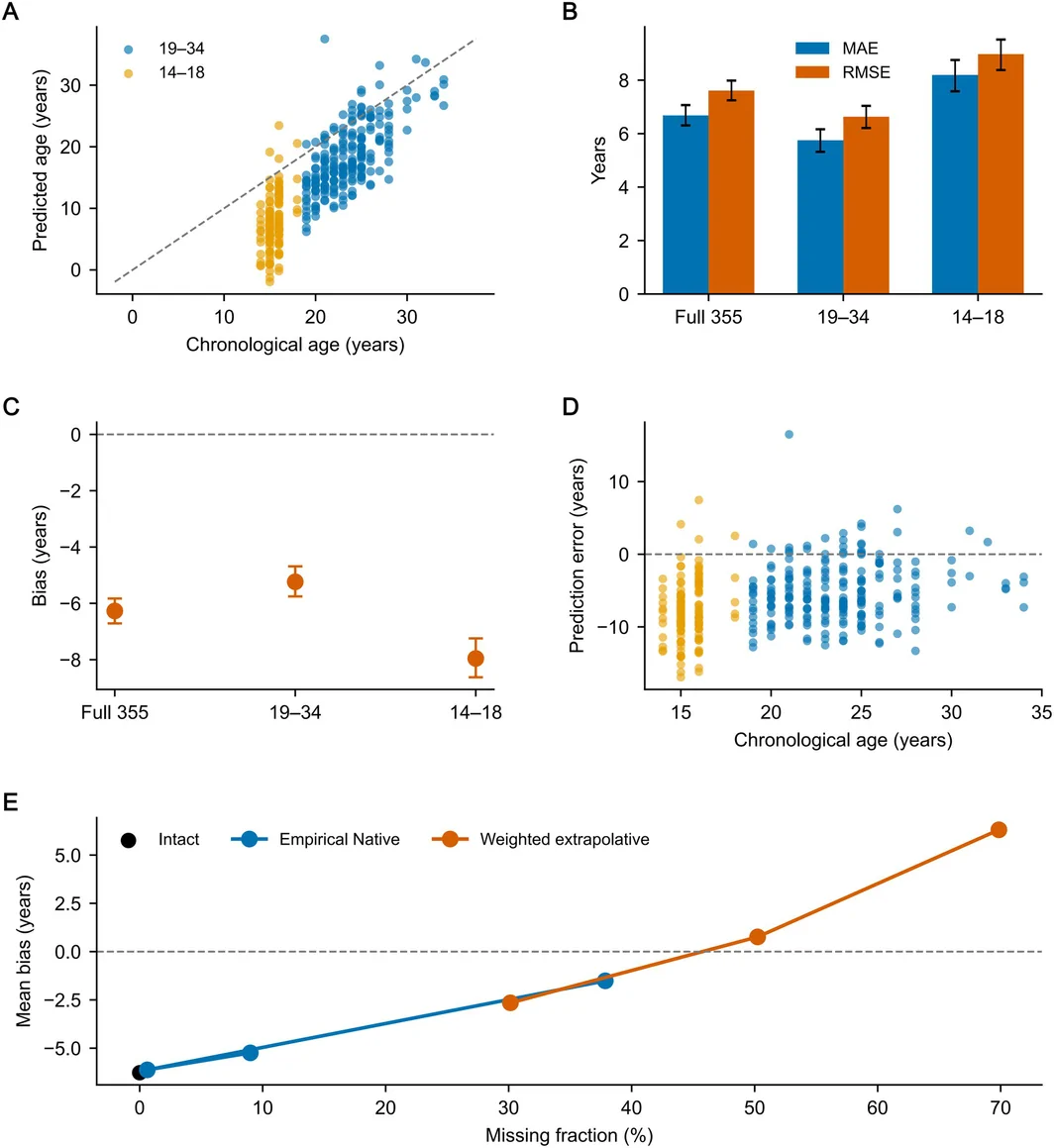
External transport and calibration of chronological‐age prediction in a younger cohort. The 209‐CpG KNN5‐RidgeCV predictor was applied to GSE73103 (*n* = 355) without refitting or recalibration. (A) Predicted versus chronological age in participants aged 19–34 years (*n* = 220) and 14–18 years (*n* = 135). The dashed line denotes identity. (B) Mean absolute error (MAE) and root‐mean‐square error (RMSE) for the full cohort and the two age strata. (C) Mean prediction bias, calculated as predicted minus chronological age. Error bars in (B) and (C) denote 95% percentile intervals from 2000 subject‐level bootstrap resamples. (D) Prediction residuals versus chronological age. The dashed line denotes zero residual. (E) Mean prediction bias for intact profiles and under empirical (Native) and susceptibility‐weighted masks with nominal missingness of 30%, 50%, and 70%. Masked estimates were averaged across ten mask assignments. MAE, mean absolute error; KNN5, five‐nearest‐neighbor reconstruction; RMSE, root‐mean‐square error.

Mean prediction bias was −6.270 years in the full cohort, −5.235 years at ages 19–34 years, and −7.958 years at ages 14–18 years (Figure 5C). The age‐on‐prediction slope was 0.539 in the full cohort and 0.0675 in the younger stratum, indicating substantial calibration bias despite an overall Pearson correlation of 0.835. Individual residuals confirmed progressively stronger underestimation below the development age range, and Pearson r within the 14–18‐year stratum was 0.368 (Figure 5D). These findings support interpreting GSE73103 as an age‐restricted transport analysis rather than as full‐lifespan external validation.

Introducing technical missingness altered the direction and magnitude of bias without resolving the underlying transport limitation. Mean bias changed from −6.270 years for intact profiles to −6.122 under S1 Native, −5.243 under ALL42 Native, and −1.512 under S4 Native masks; under susceptibility‐weighted stress conditions, bias was −2.652 years at 30%, +0.762 years at 50%, and +6.307 years at 70% missingness (Figure 5E). The corresponding RMSE trajectory is shown in Supplementary Figure 9B. This non‐monotonic pattern is consistent with an interaction between cohort differences and reference‐based reconstruction and should not be interpreted as an improvement caused by measurement loss.

### 2.7 Background‐Estimation Error Limits Recovery as Mixture Asymmetry Increases

We finally examined whether age prediction could be recovered from increasingly asymmetric mixtures when the background profile had to be estimated. At target fractions *α* = 0.50, 0.25, and 0.10, corresponding to background‐to‐target ratios of 1:1, 3:1, and 9:1, linear subtraction amplifies measurement noise by 1/α (2‐, 4‐, and 10‐fold) and background‐estimation error by (1‐α)/α (1‐, 3‐, and 9‐fold) (Figure 6A). The true personalized background was used only as an idealized reference. The evaluated estimated‐background procedure instead used the outer‐fold training set mean and converted recovered beta values outside [−0.1, 1.1] to missing before KNN5 reconstruction.

**FIGURE 6:**
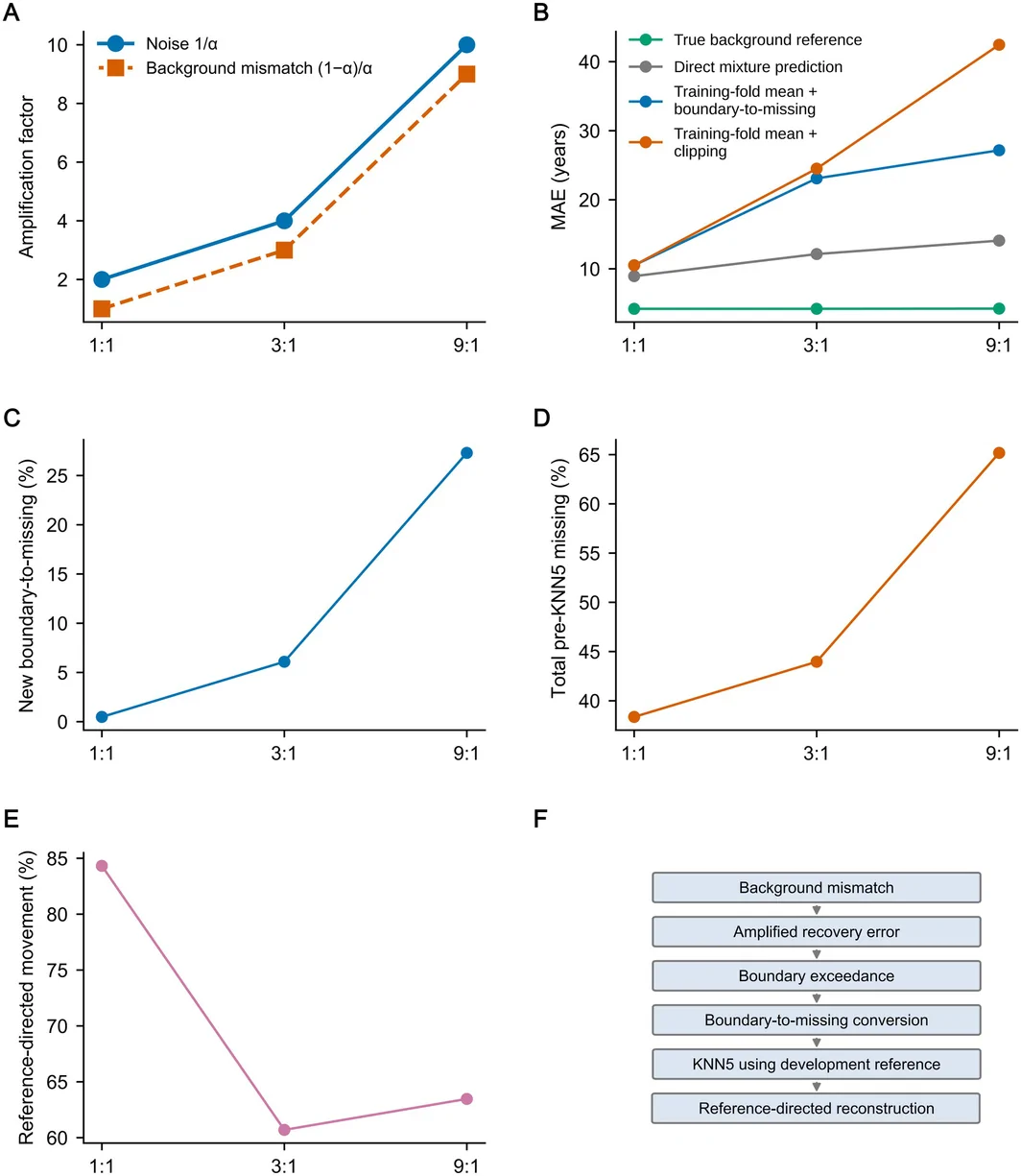
Mixture recovery under background uncertainty. (A) Algebraic amplification of measurement noise (1/α) and background mismatch [(1 − α)/α] across background‐to‐target ratios of 1:1, 3:1, and 9:1. (B) Mean absolute error (MAE) for the true‐background reference, direct mixture prediction, training‐fold mean background with boundary‐to‐missing conversion followed by KNN5 reconstruction, and training‐fold mean background with clipping. (C) Fraction of sample‐by‐CpG entries newly converted to missing by the boundary‐to‐missing rule. (D) Total missing fraction before KNN5 reconstruction, combining pre‐existing and boundary‐created missingness. (E) Fraction of boundary‐created missing entries reconstructed toward the development reference. (F) Schematic of the evaluated sequence from background mismatch and algebraic amplification to boundary‐to‐missing conversion and reference‐directed reconstruction. Panels B–E were evaluated under S4 empirical (Native) missingness with Gaussian measurement noise (standard deviation = 0.02 times the development‐fold feature standard deviation); values are means across 100 scenarios. Ratios indicate background to target. KNN5, five‐nearest‐neighbor reconstruction; MAE, mean absolute error.

Under severe S4 Native missingness with added Gaussian measurement noise (standard deviation, 0.02 times the development‐fold feature standard deviation), recovery remained stable when the true background was supplied: mean MAE was 4.210, 4.211, and 4.238 years at 1:1, 3:1, and 9:1, respectively. Replacing that background with the training‐fold mean markedly increased error. With boundary‐to‐missing conversion followed by KNN5, MAE increased to 10.512, 23.097, and 27.165 years and RMSE to 13.200, 28.316, and 32.606 years across the same mixture ratios. Direct mixture prediction yielded MAE values of 8.940, 12.138, and 14.085 years, whereas clipping after training‐mean‐background recovery yielded 10.508, 24.507, and 42.444 years (Figure 6B). Thus, neither clipping nor KNN5 compensated for the error introduced by an increasingly mismatched background estimate.

The boundary rule itself created progressively more missing values as mixture asymmetry increased: 0.483% of all sample‐by‐CpG entries at 1:1, 6.089% at 3:1, and 27.299% at 9:1 (Figure 6C). After these entries were combined with pre‐existing S4 missingness, total missingness entering KNN5 rose to 38.359%, 43.964%, and 65.174%, respectively (Figure 6D). Among entries made missing only by the boundary conversion, 84.323%, 60.698%, and 63.467% were reconstructed toward the development reference at 1:1, 3:1, and 9:1 (Figure 6E). The resulting sequence links background mismatch and algebraic amplification to boundary conversion and subsequent reference‐directed reconstruction (Figure 6F).

This sensitivity pattern persisted across intact profiles without added noise, intact profiles with noise, ALL42 Native missingness with noise, and S4 Native missingness with noise (Supplementary Figure 10A–D). Replacing the personalized background with the training‐fold mean produced substantial error even in intact profiles without added noise, indicating that background mismatch alone was sufficient to destabilize recovery. Error increased further as mixture asymmetry intensified and was largest at 9:1 under the evaluated procedure.

## 3 Discussion

This study evaluated a 209‐CpG whole‐blood chronological‐age predictor under empirical and random missingness. In the held‐out GSE40279 samples, KNN5 reconstruction reduced RMSE by 0.365 years relative to development‐set mean replacement under ALL42 Native masks (95% bootstrap interval, −0.546 to −0.205). The difference increased at higher missingness burdens, but absolute prediction error also increased. Feature membership became less stable as missingness increased, external application showed systematic age underestimation, and mixture recovery became unstable when the background was estimated under increasing asymmetry. The principal result is therefore conditional: local reconstruction lowered prediction error under the evaluated missingness patterns, but it did not remove error associated with severe missingness, cohort shift, or inaccurate background estimation.

The study separates three questions often conflated in missing‐data prediction: which CpGs are retained under perturbation, how missing CpGs are reconstructed, and how the reconstructed representation is mapped to age. The 750‐condition analysis characterized feature membership; fixed‐panel comparisons isolated reconstruction and regression; and the factorial analysis quantified component effects in a distinct selection setting. Expanded regularization removed the elastic‐net boundary issue without materially changing aggregate performance. Ridge behavior was representation dependent, with lower‐bound solutions in the all‐CpG/fold‐selected factorial spaces but interior positive penalties for the fixed 209‐CpG panel. XGBoost was evaluated as a fixed comparator rather than exhaustively optimized.

The 209‐CpG panel should be interpreted as an empirically stable predictive representation within the development analyses, not as a uniquely identifiable molecular network. The 52‐CpG subset reflects more stringent membership stability, whereas exact overlap among selected features declined with increasing missingness and three primary CpGs showed at least one coefficient‐direction reversal. Such behavior is compatible with correlation among methylation features (Teschendorff and Relton 2018). Targeted post‐KNN5 value permutation produced substantially larger losses than matched random permutation, while structural deletion followed by refitting produced smaller effects, consistent with partial compensation by correlated predictors. cg16867657 in ELOVL2 ranked highly across folds and is concordant with established chronological‐age associations (Garagnani et al. 2012; Park et al. 2016; Weidner et al. 2014; Zbieć‐Piekarska et al. 2015a, 2015b) and inter‐laboratory evidence (Garali et al. 2020). These observations concern predictive dependence within the fitted model and do not establish biological causality.

The empirical missingness templates captured recurrent and nonuniform detection‐P failures, consistent with variable FFPE array performance (de Ruijter et al. 2015; Do and Dobrovic 2015; Kling et al. 2017; Lechner et al. 2013; Siegel et al. 2014). Native masks were not consistently more damaging than matched‐count MCAR (Figure 2B); they complement random controls with observed failure patterns. When every sample and CpG failure count was preserved by randomization, KNN5 retained lower error than mean replacement, while an additional co‐failure effect was not detected. Intervals that include zero do not establish equivalence, and fixed‐marginal randomization is not MCAR. The technical templates originated from tumors rather than whole blood, and only binary failure identities were transferred. Tissue composition, pathology, restoration, and processing may therefore influence the observed patterns. In addition, the co‐failure modules do not encode genomic proximity, and neither these modules nor the constructed stress tests validate GC‐, chromatin‐, or nucleosome‐dependent physical degradation.

The held‐out analysis estimates the effect of reconstruction for a fixed predictor under prespecified masks. KNN5 changed RMSE little under S1 Native masks but reduced RMSE by 0.365 years under ALL42 and 1.759 years under S4 relative to development‐set mean replacement; the corresponding difference was 3.587 years at 70% susceptibility‐weighted missingness. These findings are consistent with nearest‐neighbor reconstruction drawing information from similar reference profiles (Dong and Peng 2013; Stekhoven and Bühlmann 2012; Troyanskaya et al. 2001; Waljee et al. 2013). Importantly, lower error relative to mean replacement did not imply restoration of intact‐profile accuracy: RMSE increased from 5.381 years for intact profiles to 5.533 under ALL42, 5.941 under S4, and 7.532 at 70% missingness.

External transport exposed a different limitation. Predictions in GSE73103 remained associated with chronological age but underestimated age by 6.27 years on average, with the largest errors among participants aged 14–18 years. This divergence between association and absolute accuracy illustrates the need to consider error, bias, and calibration jointly (Stevens and Poppe 2020; Van Calster et al. 2019). GSE40279 spans ages 19–101 years, whereas GSE73103 includes healthy individuals aged 14–34 years (Hannum et al. 2013; Voisin et al. 2015); age distribution and cohort context may therefore contribute to the shift. Because the model was applied without recalibration, these results represent direct transport of the development predictor. The non‐monotonic change in bias after masking may reflect interaction with reference‐based reconstruction and should not be interpreted as a beneficial effect of measurement loss.

Mixture recovery was dominated by background specification. Subtraction using the true personalized background produced relatively stable predictions, whereas substitution of the training‐fold mean produced large errors that increased with mixture asymmetry. This behavior follows directly from amplification of background‐estimation error by (1‐α)/α and is consistent with the broader difficulty of methylation deconvolution when source composition is uncertain (Houseman et al. 2012; Moss et al. 2018; Snyder et al. 2016). Boundary‐to‐missing conversion removed extreme recovered beta values but simultaneously increased missingness, after which KNN5 often moved the reconstructed values toward the development reference. Under the evaluated training‐mean‐background procedure, 9:1 recovery therefore had poor accuracy.

Several limitations define the scope of these results. First, the complete 750‐condition selection procedure was not repeated within outer training folds, so the holdout evaluates a fixed predictor rather than the full development procedure. Second, GSE73103 does not span the development age range, and age‐balanced external validation remains necessary. Third, prospective whole‐blood degradation experiments are needed to separate degradation, restoration, platform, and imputation effects (de Ruijter et al. 2015; Kling et al. 2017; Siegel et al. 2014). Fourth, component contrasts remain conditional on their feature representations; the fixed‐209 Ridge sensitivity was development‐only. Fifth, bootstrap intervals exclude uncertainty from panel selection, model fitting, new technical templates, and new randomization chains. Finally, biological‐age acceleration, disease risk, and clinical utility were not evaluated and require distinct outcomes and validation strategies (Belsky et al. 2020; Levine et al. 2018; Lu et al. 2019; Oblak et al. 2021).

## 4 Methods

### 4.1 Datasets, Methylation Matrices, and Cohort Roles

Age modeling used processed whole‐blood beta‐value matrices from GSE40279 and GSE73103 generated on the Illumina HumanMethylation450 platform (Bibikova et al. 2011; Hannum et al. 2013; Voisin et al. 2015). GSE40279 contained 656 profiles and GSE73103 contained 355; their ordered intersection comprised 397,278 CpGs. GSE40279 was split once (seed 20,260,829) by age‐decile‐by‐sex stratification into 524 development and 132 held‐out samples. GSE73103 was reserved for transport analysis as the full cohort and prespecified 19–34‐year (*n* = 220) and 14–18‐year (*n* = 135) strata. Sex was not used as a predictor.

### 4.2 Feature‐Stability Analysis and CpG Panel Definition

Feature selection was restricted to the 524 development samples. Elastic net (Zou and Hastie 2005) was evaluated across ten degradation seeds, five l1 ratios, five cross‐validation partition seeds, and mean, median, or exact KNN5 preprocessing, yielding 750 conditions. A 50% synthetic MCAR perturbation was used only to evaluate membership stability. Integrated criteria across seeds, preprocessing methods, and l1 settings defined the 209‐CpG primary panel and 52‐CpG stringent subset. The held‐out GSE40279 and all GSE73103 samples were excluded. Because perturbed matrices were imputed before elastic‐net cross‐validation, this analysis characterizes membership stability rather than full‐pipeline generalization.

### 4.3 Empirical Structured Missingness and Technical‐Susceptibility Analyses

Technical‐reference datasets supplied detection‐failure information only. GSE38266 provided 42 FFPE head‐and‐neck squamous cell carcinoma profiles, the sole source of ALL42 templates, susceptibility weights, and co‐failure modules. GSE38268 supplied six fresh‐frozen profiles from the same study as descriptive comparators. GSE59038 comprised 24 colon‐tumor profiles from three donors (21 FFPE across repair conditions; three fresh‐frozen); GSE92577 was the 450 K pediatric brain‐tumor subseries (three FFPE; nine fresh‐frozen). FFPE profiles from GSE59038, GSE38266, and GSE92577 informed cross‐dataset failure‐recurrence annotations; fresh‐frozen profiles provided descriptive comparisons. Only failure information, not tumor beta values or disease labels, was transferred. All 42 templates, retained without performance‐based selection, were categorized as S1 (< 1%), S2 (1%– < 5%), S3 (5%– < 20%), and S4 (> = 20%). Native masks preserved empirical failure identity and co‐failure structure; column‐permuted masks disrupted identity while preserving burden and recurrence; matched‐count MCAR masks preserved each sample's missing count. Susceptibility‐weighted stress masks removed exactly 63, 105, and 146 of 209 CpGs at nominal 30%, 50%, and 70% loss. Constructed missingness and fixed‐marginal randomization analyses are detailed in Supplementary Methods 2 and 3.

### 4.4 Component Analysis and Predictor Specification

Fixed‐panel comparisons used five‐fold predictions with preprocessing fitted within each training fold and were conditional on the predefined 209‐CpG panel. The held‐out predictor was fitted once to the full development set with Ridge α = 2.154434690031882 and retained unchanged. A separate development‐only 2 × 2 × 2 factorial analysis crossed feature space, reconstruction, and regression across five outer folds. A Ridge sensitivity analysis then compared expanded‐grid Ridge with the OLS limit in all‐CpG and fixed‐209 representations using the same outer folds; no held‐out or external samples were used. Paired OOF contrasts used 5000 subject‐level bootstrap resamples with Bonferroni adjustment across four prespecified contrasts (Supplementary Methods 1).

### 4.5 Targeted, Random, and Dose–Response Perturbation

Feature perturbation used five‐fold out‐of‐fold predictions in the development cohort. Importance was defined within each training fold as the absolute Ridge coefficient multiplied by the feature standard deviation. Structural deletion removed the top 1%, 5%, 10%, 20%, or 40% of the panel and repeated reconstruction and Ridge fitting; bottom‐ranked deletions and 200 random sets were comparators. Post‐KNN5 value permutation altered reconstructed values without refitting and was also compared with 200 random sets. These analyses quantify predictive dependence and redundancy rather than biological causality; statistical details are in Supplementary Methods 4.

### 4.6 Held‐Out Internal Evaluation

The 132‐sample GSE40279 holdout was evaluated after model specification. The prespecified primary endpoint was the mean across ten ALL42 Native masks of RMSE (KNN5‐Ridge) minus RMSE obtained with the same Ridge coefficients after development‐set mean replacement. Intact performance, S1 and S4 masks, matched‐count MCAR, and 30%, 50%, and 70% susceptibility‐weighted stress tests were secondary analyses. No model parameter or evaluation rule was updated using holdout outcomes.

### 4.7 External Transport Evaluation

The fixed predictor was applied to GSE73103 (*n* = 355) without refitting or recalibration, using the 524‐sample GSE40279 development cohort as the KNN reference. Prespecified analyses included the full cohort and the 19–34‐year and 14–18‐year strata, with secondary missingness analyses using the same mask families. Results were interpreted as age‐restricted transport rather than full‐lifespan external validation.

### 4.8 Mixture‐Recovery Stress Tests

Development‐only mixture analyses paired target and background profiles without using age or sex. Mixtures were defined as *M* = (1‐*α*)*B* + *α S* + *ε*, with *α* = 0.50, 0.25, and 0.10 corresponding to 1:1, 3:1, and 9:1 background‐to‐target ratios. Conditions included direct‐mixture prediction, true‐background recovery as an idealized reference, and training‐mean‐background recovery with boundary‐to‐missing conversion followed by KNN5 or with clipping. Gaussian noise, when included, had CpG‐specific standard deviation equal to 0.02 times the development‐fold standard deviation.

### 4.9 Statistical Analysis

MAE and RMSE summarized prediction error; correlations summarized association, and mean bias with age‐on‐prediction intercept and slope described calibration‐related behavior. Development contrasts used prespecified sign‐flip, enrichment, and bootstrap procedures with multiplicity correction where specified. Internal and external uncertainty used 2000 subject‐level percentile bootstrap replicates. Factorial and fixed‐marginal analyses used 5000 paired resamples with their Bonferroni families; the three‐mechanism comparison used 2000 paired resamples without multiplicity adjustment. Feature‐perturbation *p* values and *q*‐value families are detailed in Supplementary Methods 4.

### 4.10 Software Environments

Analyses used Python 3.12 with NumPy, pandas, SciPy, scikit‐learn, XGBoost, matplotlib, and related packages. Full software versions and stage‐specific dependencies are provided in the Supporting Information S1 and analysis code.

## Author Contributions

X.Z., Y.L., B.H., S.W., and Y.Z. conceptualized the study. X.Z., Z.W., T.Y., and R.Z. developed the methodology. Z.W., T.Y., R.Z., and S.Y. contributed software and visualization. X.Z., Y.L., and B.H. validated the results. Z.W., T.Y., R.Z., and S.Y. performed formal analysis. X.Z., Y.L., and B.H. conducted the investigation. S.W., Y.Z., and X.Z. provided resources, supervision, and project administration. Z.W., T.Y., R.Z., and S.Y. curated the data. X.Z., S.W., and Y.Z. acquired funding. X.Z. drafted the manuscript. All authors reviewed and edited the manuscript and approved the final version.

## Funding

This work was supported by grants from the National Natural Science Foundation of China (32100826), the China Postdoctoral Science Foundation (2022M7107), and the Fujian Provincial Natural Science Foundation (2025J01502, 2026J001234).

## Ethics Statement

This study reanalyzed publicly available, de‐identified methylation datasets and did not recruit new participants or collect new biospecimens.

## Conflicts of Interest

The authors declare no conflicts of interest.

## Supporting information


**Supplementary Figure 1.** Selection stability of the 209‐CpG panel. (A) Selection fractions across 750 prespecified development conditions spanning missingness seeds, cross‐validation partitions, elastic‐net mixing parameters, and preprocessing strategies in the 524‐sample development set. CpGs are ordered by selection fraction; orange points denote the 52‐CpG stringent sensitivity subset. (B) Selection fractions for individual CpGs across elastic‐net l1 ratios of 0.1, 0.3, 0.5, 0.7, and 0.9. Color denotes selection fraction.
**Supplementary Figure 2**. Missingness‐related performance and feature stability across preprocessing strategies. (A) Difference in root‐mean‐square error (RMSE) between empirical (Native) and matched‐count MCAR masks (Native minus MCAR). (B) Median pairwise Jaccard similarity of CpG sets selected across ten mask assignments under empirical (Native) masks. Both analyses compare development‐set mean replacement, median replacement, KNN5 reconstruction, and exclusion of the core or broad technical‐vulnerability sets (TV_CORE or TV_BROAD) in the 524‐sample development set. S1, < 1% missingness; S4, ≥ 20% missingness; ALL42, all 42 FFPE‐derived templates; FFPE, formalin‐fixed paraffin‐embedded; KNN5, five‐nearest‐neighbor reconstruction; MCAR, missing completely at random.
**Supplementary Figure 3**. CpG‐panel size sensitivity and importance of cg16867657. (A) Difference in root‐mean‐square error (RMSE) between the 52‐CpG stringent sensitivity subset and the 209‐CpG primary panel using Ridge regression with development‐set mean replacement (52‐CpG minus 209‐CpG). Estimates were averaged across ten mask assignments for S1, ALL42, and S4 empirical (Native), column‐permuted, and matched‐count MCAR masks. (B) Importance rank of cg16867657 (ELOVL2) in each of five outer training folds, calculated as the absolute Ridge coefficient multiplied by the training‐fold feature standard deviation. Analyses used the 524‐sample development set. S1, < 1% missingness; S4, ≥ 20% missingness; ALL42, all 42 FFPE‐derived templates; MCAR, missing completely at random.
**Supplementary Figure 4**. Factorial comparison of feature space, reconstruction, and regression under expanded regularization ranges. (A) Mean absolute error (MAE) for eight combinations of feature space (397,278 common CpGs or fold‐specific elastic‐net selection), reconstruction (development‐set mean replacement or KNN5), and regression (RidgeCV or XGBoost). (B) Main effects, pairwise interactions, and the three‐way interaction in MAE. Main effects compare selected with all CpGs, KNN5 with mean replacement, and XGBoost with Ridge, averaged across the remaining factors. Points denote effect estimates; lines denote Bonferroni‐adjusted percentile intervals across seven effects from 5000 paired subject‐level bootstrap resamples. (C) Ridge and XGBoost MAE after KNN5 reconstruction in each feature space; Δ denotes XGBoost minus Ridge. Estimates were averaged across ten ALL42 empirical (Native) mask assignments in the 524‐sample development set evaluated in five outer folds. Expanded elastic‐net selection retained 3093–3243 CpGs per fold. ALL42, all 42 FFPE‐derived templates; EN, elastic net; KNN5, five‐nearest‐neighbor reconstruction.
**Supplementary Figure 5**. Reconstruction performance across three missingness mechanisms. (A, C, E) Mean absolute error (MAE) and (B, D, F) root‐mean‐square error (RMSE) under co‐failure, susceptibility‐weighted, and exact‐count MCAR masks, respectively. Blue circles denote KNN5 reconstruction and vermilion squares denote development‐set mean replacement. Large markers denote means across ten mask assignments, small jittered points denote individual assignments, and dotted lines denote intact‐profile error. Nominal missingness of 30%, 50%, and 70% corresponds to 63, 105, and 146 of the 209 CpGs per sample, respectively. (G, H) KNN5‐minus‐mean differences in MAE and RMSE, respectively. Diamonds denote mean paired differences; horizontal lines denote 95% percentile intervals from 2000 paired subject‐level bootstrap resamples. C, S, and M denote co‐failure, susceptibility‐weighted, and MCAR masks, respectively. Analyses used five‐fold out‐of‐fold predictions in the 524‐sample development set. KNN5, five‐nearest‐neighbor reconstruction; MCAR, missing completely at random.
**Supplementary Figure 6**. Prediction‐error responses to targeted and comparator feature perturbations. (A) Change in root‐mean‐square error (ΔRMSE) across structural‐deletion doses for top‐ranked CpGs and the mean of 200 random deletion sets under intact, ALL42 Native, and S4 Native conditions. (B) ΔRMSE across structural‐deletion doses comparing top‐ranked with bottom‐ranked CpG deletion under the same three conditions. (C) ΔRMSE across post‐KNN5 feature‐value permutation doses for top‐ranked CpGs and the mean of 200 random‐feature permutation sets under intact, ALL42 Native, and S4 Native conditions. (D) Importance rank of cg16867657 (ELOVL2) across five outer cross‐validation folds; numbers indicate the fold‐specific ranks. In (A–C), top del denotes top‐ranked structural deletion, rand del denotes the mean across 200 random deletion sets, bottom del denotes bottom‐ranked structural deletion, top perm denotes post‐KNN5 value permutation of top‐ranked CpGs, and rand perm denotes the mean across 200 random‐feature permutation sets. Solid lines with circles denote top‐ranked perturbations; dashed lines with squares denote random‐set means in (A) and (C); dotted lines with inverted triangles denote bottom‐ranked deletion in (B). Green, blue, and vermilion lines denote intact, ALL42 Native, and S4 Native conditions, respectively. ΔRMSE was calculated as RMSE after perturbation minus the RMSE of the corresponding unperturbed 209‐CpG baseline within the same context. Feature ranking was based on the absolute Ridge coefficient multiplied by the training‐fold feature standard deviation. Perturbation doses of 1%, 5%, 10%, 20%, and 40% correspond to 2, 10, 21, 42, and 84 CpGs. Structural deletion includes re‐imputation and Ridge refitting, whereas post‐KNN5 permutation alters reconstructed feature values without repeating imputation or refitting. ALL42, all 42 empirical failure templates; S4, templates with ≥ 20% missingness; KNN5, five‐nearest‐neighbor reconstruction.
**Supplementary Figure 7**. Targeted perturbation effects across missingness conditions. (A) Targeted‐minus‐random change in root‐mean‐square error (ΔRMSE) after structural deletion with re‐imputation and Ridge refitting. (B) Targeted‐minus‐random ΔRMSE after permutation of reconstructed feature values with the fitted predictor retained. Perturbation doses of 1%, 5%, 10%, 20%, and 40% correspond to 2, 10, 21, 42, and 84 of the 209 CpGs. Analyses used five‐fold predictions in the 524‐sample development set, with one intact realization and ten mask assignments for each masked condition. S1, < 1% missingness; S4, ≥ 20% missingness; ALL42, all 42 FFPE‐derived templates; Native, empirical probe‐failure pattern; MCAR, missing completely at random; KNN5, five‐nearest‐neighbor reconstruction.
**Supplementary Figure 8**. Changes in mean absolute error after feature perturbation. (A, B) Intact profiles. (C, D) ALL42 empirical (Native) masks. (E, F) S4 empirical (Native) masks. (G, H) S1 empirical (Native) masks. (I, J) ALL42 matched‐count MCAR masks. (K, L) S4 matched‐count MCAR masks. Panels A, C, E, G, I, and K show structural deletion with re‐imputation and Ridge refitting; panels B, D, F, H, J, and L show permutation of reconstructed feature values with the fitted predictor retained. ΔMAE is defined as perturbed minus baseline mean absolute error. Blue circles denote targeted perturbation, vermilion triangles denote bottom‐ranked deletion, and gray squares denote means across 200 random feature sets; gray shading denotes the 2.5th–97.5th percentiles of the random‐set distribution. Perturbation doses of 1%, 5%, 10%, 20%, and 40% correspond to 2, 10, 21, 42, and 84 of the 209 CpGs. Analyses used five‐fold predictions in the 524‐sample development set. S1, < 1% missingness; S4, ≥ 20% missingness; ALL42, all 42 FFPE‐derived templates; KNN5, five‐nearest‐neighbor reconstruction; MCAR, missing completely at random.
**Supplementary Figure 9**. Prediction error across missingness burdens in the internal and external cohorts. (A) Root‐mean‐square error (RMSE) versus observed missing fraction in the GSE40279 held‐out internal set (*n* = 132). (B) RMSE versus observed missing fraction in GSE73103 (*n* = 355). Black diamonds denote intact profiles, blue circles denote empirical S1, ALL42, and S4 Native masks, and vermilion squares denote susceptibility‐weighted masks with nominal missingness of 30%, 50%, and 70%. Masked estimates were averaged across ten mask assignments. The 209‐CpG KNN5‐RidgeCV predictor was applied without refitting or recalibration. S1, < 1% missingness; S4, ≥ 20% missingness; ALL42, all 42 FFPE‐derived templates; KNN5, five‐nearest‐neighbor reconstruction.
**Supplementary Figure 10**. Mixture‐recovery accuracy across missingness and noise conditions. (A) Intact profiles without added noise. (B) Intact profiles with measurement noise. (C) ALL42 empirical (Native) missingness with measurement noise. (D) S4 empirical (Native) missingness with measurement noise. Mean absolute error (MAE) was evaluated at background‐to‐target ratios of 1:1, 3:1, and 9:1 for true‐background recovery, direct mixture prediction, and recovery using the training‐fold mean background with boundary‐to‐missing conversion or clipping. Gaussian noise was generated with a standard deviation equal to 0.02 times the training‐fold feature standard deviation. Points denote mean MAE across ten simulation scenarios for intact profiles and 100 scenarios for masked profiles. Boundary‐to‐missing conversion treats recovered beta values outside [−0.1, 1.1] as missing before KNN5 reconstruction. Analyses used the 524‐sample development set. ALL42 denotes all 42 FFPE‐derived templates; S4 denotes templates with ≥ 20% missingness; KNN5, five‐nearest‐neighbor reconstruction.

**Data S1:** acel70800‐sup‐0002‐supinfo.docx. **Supplementary Methods 1**. Factorial comparison and parameter‐range sensitivity.
**Supplementary Methods 2**. Reconstruction under three constructed missingness mechanisms.
**Supplementary Methods 3**. Randomization with fixed sample and CpG failure counts.
**Supplementary Methods 4**. Feature perturbation analyses.

**Supplementary Table 1.** Factorial effects of feature space, reconstruction, and regression across regularization ranges.

**Supplementary Table 2.** Reconstruction performance under co‐failure‐module, susceptibility‐weighted, and exact‐count MCAR missingness.

**Supplementary Table 3.** Reconstruction performance under co‐failure masks and fixed‐marginal randomization.

**Supplementary Table 4.** Feature‐perturbation effects across six missingness contexts, five perturbation doses, and two perturbation types.

## Data Availability

Methylation matrices are available from GEO under GSE40279 and GSE73103; technical‐reference datasets are GSE59038, GSE38266, GSE38268, and GSE92577. Supplementary result tables, CpG and sample‐order specifications, fitted Ridge coefficients, development‐set feature means, and analysis and model‐export scripts accompany the revision. The complete 524 × 209 development‐reference beta matrix required for KNN5 reconstruction is not included in the supplied model files. The GEO data and supplied ordering specifications identify the inputs needed to reconstruct this reference matrix, but the coefficient and mean files alone do not constitute a self‐contained predictor for incomplete profiles. Analysis code is publicly available at https://github.com/tenfeum/AgingCell‐Methylation‐Age‐Missingness_v1.0.0.
