# Supplementary material for: A 209‐Site DNA Methylation Predictor of Chronological Age Evaluated Under Empirical and Random Missingness
Source: Aging Cell. 2026 Nov 1;25(11):e70800. doi: 10.1111/acel.70800 (PMC13724237; doi:10.1111/acel.70800)
Supplement: Supplementary file 1 [file ACEL-25-e70800-s001.docx]

**Supplementary Figures**

**Supplementary Figure 1. Selection stability of the 209-CpG panel.**

(A) Selection fractions across 750 prespecified development conditions spanning missingness seeds, cross-validation partitions, elastic-net mixing parameters, and preprocessing strategies in the 524-sample development set. CpGs are ordered by selection fraction; orange points denote the 52-CpG stringent sensitivity subset.

(B) Selection fractions for individual CpGs across elastic-net l1 ratios of 0.1, 0.3, 0.5, 0.7, and 0.9. Color denotes selection fraction.

**Supplementary Figure 2. Missingness-related performance and feature stability across preprocessing strategies.**

(A) Difference in root-mean-square error (RMSE) between empirical (Native) and matched-count MCAR masks (Native minus MCAR).

(B) Median pairwise Jaccard similarity of CpG sets selected across ten mask assignments under empirical (Native) masks.

Both analyses compare development-set mean replacement, median replacement, KNN5 reconstruction, and exclusion of the core or broad technical-vulnerability sets (TV_CORE or TV_BROAD) in the 524-sample development set. S1, <1% missingness; S4, ≥20% missingness; ALL42, all 42 FFPE-derived templates; KNN5, five-nearest-neighbor reconstruction; FFPE, formalin-fixed paraffin-embedded; MCAR, missing completely at random.

**Supplementary Figure 3. CpG-panel size sensitivity and importance of cg16867657.**

(A) Difference in root-mean-square error (RMSE) between the 52-CpG stringent sensitivity subset and the 209-CpG primary panel using Ridge regression with development-set mean replacement (52-CpG minus 209-CpG). Estimates were averaged across ten mask assignments for S1, ALL42, and S4 empirical (Native), column-permuted, and matched-count MCAR masks.

(B) Importance rank of cg16867657 (ELOVL2) in each of five outer training folds, calculated as the absolute Ridge coefficient multiplied by the training-fold feature standard deviation.

Analyses used the 524-sample development set. S1, <1% missingness; S4, ≥20% missingness; ALL42, all 42 FFPE-derived templates; MCAR, missing completely at random.

**Supplementary Figure 4. Factorial comparison of feature space, reconstruction, and regression under expanded regularization ranges.**

(A) Mean absolute error (MAE) for eight combinations of feature space (397,278 common CpGs or fold-specific elastic-net selection), reconstruction (development-set mean replacement or KNN5), and regression (RidgeCV or XGBoost).

(B) Main effects, pairwise interactions, and the three-way interaction in MAE. Main effects compare selected with all CpGs, KNN5 with mean replacement, and XGBoost with Ridge, averaged across the remaining factors. Points denote effect estimates; lines denote Bonferroni-adjusted percentile intervals across seven effects from 5,000 paired subject-level bootstrap resamples.

(C) Ridge and XGBoost MAE after KNN5 reconstruction in each feature space; Δ denotes XGBoost minus Ridge.

Estimates were averaged across ten ALL42 empirical (Native) mask assignments in the 524-sample development set evaluated in five outer folds. Expanded elastic-net selection retained 3,093–3,243 CpGs per fold. ALL42, all 42 FFPE-derived templates; EN, elastic net; KNN5, five-nearest-neighbor reconstruction.

**Supplementary Figure 5. Reconstruction performance across three missingness mechanisms.**

(A, C, E) Mean absolute error (MAE) and (B, D, F) root-mean-square error (RMSE) under co-failure, susceptibility-weighted, and exact-count MCAR masks, respectively. Blue circles denote KNN5 reconstruction and vermilion squares denote development-set mean replacement. Large markers denote means across ten mask assignments, small jittered points denote individual assignments, and dotted lines denote intact-profile error. Nominal missingness of 30%, 50%, and 70% corresponds to 63, 105, and 146 of the 209 CpGs per sample, respectively.

(G, H) KNN5-minus-mean differences in MAE and RMSE, respectively. Diamonds denote mean paired differences; horizontal lines denote 95% percentile intervals from 2,000 paired subject-level bootstrap resamples. C, S, and M denote co-failure, susceptibility-weighted, and MCAR masks, respectively. Analyses used five-fold out-of-fold predictions in the 524-sample development set. KNN5, five-nearest-neighbor reconstruction; MCAR, missing completely at random.

**Supplementary Figure 6. Prediction-error responses to targeted and comparator feature perturbations.**

(A) Change in root-mean-square error (ΔRMSE) across structural-deletion doses for top-ranked CpGs and the mean of 200 random deletion sets under intact, ALL42 Native, and S4 Native conditions.

(B) ΔRMSE across structural-deletion doses comparing top-ranked with bottom-ranked CpG deletion under the same three conditions.

(C) ΔRMSE across post-KNN5 feature-value permutation doses for top-ranked CpGs and the mean of 200 random-feature permutation sets under intact, ALL42 Native, and S4 Native conditions.

(D) Importance rank of cg16867657 (ELOVL2) across five outer cross-validation folds; numbers indicate the fold-specific ranks.

In (A–C), top del denotes top-ranked structural deletion, rand del denotes the mean across 200 random deletion sets, bottom del denotes bottom-ranked structural deletion, top perm denotes post-KNN5 value permutation of top-ranked CpGs, and rand perm denotes the mean across 200 random-feature permutation sets. Solid lines with circles denote top-ranked perturbations; dashed lines with squares denote random-set means in (A) and (C); dotted lines with inverted triangles denote bottom-ranked deletion in (B). Green, blue, and vermilion lines denote intact, ALL42 Native, and S4 Native conditions, respectively. ΔRMSE was calculated as RMSE after perturbation minus the RMSE of the corresponding unperturbed 209-CpG baseline within the same context. Feature ranking was based on the absolute Ridge coefficient multiplied by the training-fold feature standard deviation. Perturbation doses of 1%, 5%, 10%, 20%, and 40% correspond to 2, 10, 21, 42, and 84 CpGs. Structural deletion includes re-imputation and Ridge refitting, whereas post-KNN5 permutation alters reconstructed feature values without repeating imputation or refitting. ALL42, all 42 empirical failure templates; S4, templates with ≥20% missingness; KNN5, five-nearest-neighbor reconstruction.

**Supplementary Figure 7. Targeted perturbation effects across missingness conditions.**

(A) Targeted-minus-random change in root-mean-square error (ΔRMSE) after structural deletion with re-imputation and Ridge refitting.

(B) Targeted-minus-random ΔRMSE after permutation of reconstructed feature values with the fitted predictor retained.

Perturbation doses of 1%, 5%, 10%, 20%, and 40% correspond to 2, 10, 21, 42, and 84 of the 209 CpGs. Analyses used five-fold predictions in the 524-sample development set, with one intact realization and ten mask assignments for each masked condition. S1, <1% missingness; S4, ≥20% missingness; ALL42, all 42 FFPE-derived templates; Native, empirical probe-failure pattern; MCAR, missing completely at random; KNN5, five-nearest-neighbor reconstruction.

**Supplementary Figure 8. Changes in mean absolute error after feature perturbation.**

(A, B) Intact profiles.

(C, D) ALL42 empirical (Native) masks.

(E, F) S4 empirical (Native) masks.

(G, H) S1 empirical (Native) masks.

(I, J) ALL42 matched-count MCAR masks.

(K, L) S4 matched-count MCAR masks.

Panels A, C, E, G, I, and K show structural deletion with re-imputation and Ridge refitting; panels B, D, F, H, J, and L show permutation of reconstructed feature values with the fitted predictor retained. ΔMAE is defined as perturbed minus baseline mean absolute error. Blue circles denote targeted perturbation, vermilion triangles denote bottom-ranked deletion, and gray squares denote means across 200 random feature sets; gray shading denotes the 2.5th–97.5th percentiles of the random-set distribution. Perturbation doses of 1%, 5%, 10%, 20%, and 40% correspond to 2, 10, 21, 42, and 84 of the 209 CpGs. Analyses used five-fold predictions in the 524-sample development set. S1, <1% missingness; S4, ≥20% missingness; ALL42, all 42 FFPE-derived templates; KNN5, five-nearest-neighbor reconstruction; MCAR, missing completely at random.

**Supplementary Figure 9. Prediction error across missingness burdens in the internal and external cohorts.**

(A) Root-mean-square error (RMSE) versus observed missing fraction in the GSE40279 held-out internal set (n = 132).

(B) RMSE versus observed missing fraction in GSE73103 (n = 355).

Black diamonds denote intact profiles, blue circles denote empirical S1, ALL42, and S4 Native masks, and vermilion squares denote susceptibility-weighted masks with nominal missingness of 30%, 50%, and 70%. Masked estimates were averaged across ten mask assignments. The 209-CpG KNN5-RidgeCV predictor was applied without refitting or recalibration. S1, <1% missingness; S4, ≥20% missingness; ALL42, all 42 FFPE-derived templates; KNN5, five-nearest-neighbor reconstruction.

**Supplementary Figure 10. Mixture-recovery accuracy across missingness and noise conditions.**

(A) Intact profiles without added noise.

(B) Intact profiles with measurement noise.

(C) ALL42 empirical (Native) missingness with measurement noise.

(D) S4 empirical (Native) missingness with measurement noise.

Mean absolute error (MAE) was evaluated at background-to-target ratios of 1:1, 3:1, and 9:1 for true-background recovery, direct mixture prediction, and recovery using the training-fold mean background with boundary-to-missing conversion or clipping. Gaussian noise was generated with a standard deviation equal to 0.02 times the training-fold feature standard deviation. Points denote mean MAE across ten simulation scenarios for intact profiles and 100 scenarios for masked profiles. Boundary-to-missing conversion treats recovered beta values outside [−0.1, 1.1] as missing before KNN5 reconstruction. Analyses used the 524-sample development set. ALL42 denotes all 42 FFPE-derived templates; S4 denotes templates with ≥20% missingness; KNN5, five-nearest-neighbor reconstruction.
