# Supplementary material for: A 209‐Site DNA Methylation Predictor of Chronological Age Evaluated Under Empirical and Random Missingness
Source: Aging Cell. 2026 Nov 1;25(11):e70800. doi: 10.1111/acel.70800 (PMC13724237; doi:10.1111/acel.70800)
Supplement: Supplementary file 2 [file ACEL-25-e70800-s002.docx]

**Supplementary Methods**

**Supplementary Methods 1 Factorial comparison and parameter-range sensitivity**

The factorial analysis used 524 development samples and five outer folds. The 132-sample internal holdout and GSE73103 were excluded. Within each outer training fold, a single elastic-net selector used three inner folds and l1=0.5. Each inner training partition determined its feature means, target mean, and alpha_max; outer-validation ages were excluded from selection and tuning. Features were not standardized. Convergence tolerance was 0.0001 and the iteration ceiling was 240,000. A feature was retained when its fitted coefficient was nonzero. This selector does not repeat the 750-condition stability-selection hierarchy.

The original 12 alpha/alpha_max ratios from 1 to 0.001 were retained and supplemented by eight smaller ratios down to 0.00001. The expanded selectors retained 3,093, 3,150, 3,147, 3,128, and 3,243 CpGs, choosing relative alphas 0.000031623, 0.000031623, 0.000316228, 0.000031623, and 0.000031623. Ridge retained its original candidates and extended the lower bound from 0.001 to 0.00000001; the upper bound remained 100,000,000. Tuning used leave-one-out error with the eigen-decomposition implementation of RidgeCV. All-CpG and selected-CpG Ridge models chose the new lower bound in all five folds. On the same newly selected panel, expanded-versus-original Ridge candidate comparisons improved training cross-validated MSE by 0.000230-0.000265 years squared; corresponding all-CpG improvements were 0.000039-0.000046. These diagnostics compare candidate sets on the new selected panel rather than replaying the earlier feature selection.

Eight pipelines crossed all 397,278 common CpGs versus selected features, training-mean replacement versus KNN5, and Ridge versus XGBoost. XGBoost used 1,000 rounds, maximum depth 5, learning rate 0.02, subsample and colsample_bytree of 0.8, squared-error loss, histogram tree construction, max_bin=256, min_child_weight=1, reg_alpha=0, reg_lambda=1, seed 42, and no early stopping. Evaluation comprised 41 conditions: intact and ten realizations each of ALL42 Native, ALL42 MCAR, S4 Native, and S4 MCAR. Masks were generated in the common CpG universe before projection. Within a feature space and regressor, reconstruction methods used the same fitted regressor and differed only in filling validation profiles. Fixed 209-CpG, variance-ranked, and random size-matched feature sets were additional conditional or descriptive comparisons. There was one random size-matched set per fold, not a repeated random-set null distribution.

For each mask realization, MAE and RMSE were calculated from pooled outer-fold predictions and then averaged across realizations. Seven primary ALL42 Native MAE effects comprised three main effects, three pairwise interactions, and one three-way interaction. Main effects average a component difference across the other two factors. Pairwise interactions average differences-in-differences across the third factor; the three-way term is the difference between those interactions. Intervals used 5,000 paired subject-level bootstrap resamples, with the same subjects carried across algorithms and masks, and Bonferroni tail probability 0.05/(2 × 7). Fits, folds, selected features, and masks were held fixed. Other contrasts have individual conditional intervals; algorithm-level Shapley allocations are descriptive and do not represent CpG-level SHAP values.

An additional development-only Ridge sensitivity analysis used the same five outer folds and compared the all-397,278-CpG representation with the fixed 209-CpG panel, without fold-specific feature reselection. Within each outer training fold, Ridge was evaluated over an expanded positive-alpha grid spanning 1 × 10^-12 to 1 × 10^8 together with the OLS limit (alpha=0). For the fixed 209-CpG panel, selected alphas were 2.154434690031882, 1.0, 2.154434690031882, 2.154434690031882, and 1.0 across folds 1-5, respectively; none lay at the lower boundary. Pooled OOF MAE/RMSE were 3.503/4.810 years for selected Ridge and 4.364/5.725 years for OLS. The selected-Ridge minus OLS contrasts were -0.861 years for MAE (Bonferroni-adjusted interval, -1.159 to -0.563) and -0.915 years for RMSE (-1.281 to -0.530). In the all-CpG representation, the raw positive minimizer was at the lower boundary in all five folds, but 11-12 near-zero candidates were numerically tied and Ridge and OLS OOF performance was practically identical. Intervals used 5,000 paired subject-level bootstrap resamples with Bonferroni adjustment across four prespecified Ridge-versus-OLS contrasts. The 132-sample holdout and GSE73103 were excluded from this analysis.

**Supplementary Methods 2 Reconstruction under three constructed missingness mechanisms**

The fixed 209-CpG panel was evaluated by five-fold cross-validation in the 524-sample development cohort. The 132-sample internal holdout and GSE73103 were excluded. Within each training fold, KNN5 and RidgeCV were fitted using intact profiles. The training-mean comparison used the same Ridge coefficients and intercept, so the methods differed only in reconstruction of missing validation values. These estimates are conditional on panel selection from the full development cohort.

Binary failure patterns from the 42 GSE38266 FFPE profiles were projected onto the 209 CpGs. Probe susceptibility was estimated as (failure count + 0.5)/(42 + 1). Co-failure modules were defined by average-linkage hierarchical clustering of Jaccard distances between probe-failure vectors, using a distance threshold of 0.7. This produced 74 modules, with 82 probes in the largest module.

Three mechanisms were evaluated: susceptibility-weighted sampling without replacement, co-failure-module sampling, and uniformly sampled exact-count MCAR. Module order was sampled according to mean susceptibility within each module. Whole modules were selected where possible; if a module exceeded the remaining count, the required probes were sampled uniformly within it. Nominal 30%, 50%, and 70% missingness corresponded to exactly 63, 105, and 146 CpGs per sample. Ten seeds (20262001-20262010) for each mechanism and dose yielded 90 mask realizations and 180 method-specific sets of error metrics. Each realization included paired out-of-fold predictions for all 524 development samples.

Partial module sampling occurred in 5,152/5,240 (98.32%), 4,137/5,240 (78.95%), and 3,417/5,240 (65.21%) sample-by-mask combinations at the three doses. Marginal probe-failure frequencies and within-module co-missing covariance were quantified. Marginal frequencies were not matched between mechanisms, so the comparisons do not isolate dependence alone. Co-failure modules describe joint technical failures and do not encode genomic coordinates or establish a spatial degradation mechanism.

For each mechanism and dose, the point estimate was the mean of ten KNN5-minus-training-mean error differences. Individual 95% percentile intervals used 2,000 subject-level bootstrap resamples, paired across methods and all ten mask realizations. The PCG64 random-number generator used prespecified, mechanism-specific seeds derived from base seed 20262021. Models and masks were held fixed during resampling. Intervals were not adjusted for multiple comparisons and exclude uncertainty from panel selection, model fitting, and technical-template sampling. Supplementary Table 2 reports 18 paired contrasts and 180 realization-level error summaries.

**Supplementary Methods 3 Randomization with fixed sample and CpG failure counts**

Existing co-failure masks at nominal 30%, 50%, and 70% missingness contained exactly 63, 105, and 146 missing CpGs per sample. Each burden comprised ten realizations in the 524-sample development cohort. Four separately seeded Curveball chains per realization redistributed missing positions while retaining every row and column total (Carstens, 2015, 2016; Strona et al., 2014). At each attempted trade, two distinct rows were chosen uniformly, shared missing positions were retained, and positions missing in only one row were randomly reassigned while preserving each row total. No-change reallocations and row pairs that could not change remained counted attempts; trades were not repeated until a change occurred.

Each chain ran for 40 sweeps, with 524 attempted row trades per sweep. At sweeps 10, 20, and 40, we recorded row and column margin errors, the fraction of entries differing from the starting mask, and mean absolute off-diagonal covariance. All 360 recorded states had zero margin errors. Hamming and covariance summaries were broadly stable across the recorded checkpoints, but these finite-chain summaries do not establish convergence or exact uniform sampling. Randomized masks retain heterogeneous CpG failure frequencies and dependence imposed by fixed totals; they are not MCAR.

KNN5 and mean replacement used the existing fold-specific reference data and Ridge coefficients. Each realization and chain produced its own error metric before averaging; predictions were not averaged before computing errors. Four-chain randomized-mask KNN5 MAE ranges were 3.966-4.047, 4.504-4.554, and 5.618-5.699 years at the three burdens. The fixed 209-CpG panel was defined from all development samples, so this is a conditional reconstruction comparison rather than a nested evaluation of complete panel selection.

Five contrasts at each burden compared KNN5 with mean replacement under each mask type, co-failure with randomized masks under each reconstruction method, and the difference between the two KNN5-minus-mean effects. The 15 MAE contrasts formed the primary family; 15 RMSE contrasts formed a separate secondary family. Bonferroni-adjusted percentile intervals used 5,000 paired subject-level resamples and tail probability 0.05/30 within each family. Subjects were carried together across methods, burdens, realizations, and chains. Panels, folds, fitted models, and realized masks and chains were fixed. Intervals therefore exclude variability from panel selection, fitting, new chains, and technical-template sampling. An interval containing zero does not demonstrate equivalence. Supplementary Table 3 reports metrics, contrasts, and chain diagnostics.

**Supplementary Methods 4 Feature perturbation analyses**

MAE and RMSE were evaluated using five-fold out-of-fold predictions for the 524 GSE40279 development samples. Analyses compared targeted and bottom-ranked structural deletion, post-KNN5 value permutation, and 200 matched random-feature sets per dose. The 209-CpG panel was fixed before these perturbation analyses.

Nominal doses of 1%, 5%, 10%, 20%, and 40% corresponded to 2, 10, 21, 42, and 84 deleted or permuted CpGs. Structural deletion included re-imputation and Ridge refitting within each training fold. Post-KNN5 value permutation changed reconstructed values within each validation fold without repeating imputation or refitting; feature-specific permutations were reused across doses and random sets. Intact profiles contributed one realization, and each masked context contributed ten equally weighted realizations. Intact, ALL42 Native, and S4 Native constituted the primary context tier; S1 Native, ALL42 MCAR, and S4 MCAR constituted the secondary tier.

For each realization, MAE and RMSE were calculated from pooled out-of-fold predictions. Delta denotes perturbed error minus the corresponding unperturbed error. Realization-level estimates were averaged equally within each masked context. Targeted-minus-random contrasts subtract the mean delta across 200 random feature sets from the targeted delta. One-sided empirical P values were calculated as (1 + number of random deltas at least as large as the targeted delta)/201. Benjamini-Hochberg adjustment was applied separately by metric, perturbation type, and context tier across 15 tests per family. The envelopes in Supplementary Figure 8 span the 2.5th-97.5th percentiles across random feature sets; they quantify random-set variation rather than subject-sampling uncertainty.

MAE and RMSE were calculated from the same predictions and perturbations. Supplementary Table 4 reports targeted, random, and bottom-ranked effects, together with P values and adjusted q values, for 120 comparisons.

**Software environments**

Core analyses used Python 3.12.14, NumPy 2.5.2, pandas 3.0.5, SciPy 1.18.0, scikit-learn 1.9.0, XGBoost 3.4.1, PyArrow 25.0.0, matplotlib 3.11.1, statsmodels 0.14.6, PyYAML 6.0.3, and joblib 1.5.3.

Feature perturbation, constructed missingness, and additional range and fixed-marginal analyses used Python 3.12.3, NumPy 2.3.5, pandas 2.2.3, SciPy 1.17.0, scikit-learn 1.8.0, joblib 1.5.3, threadpoolctl 3.6.0, and matplotlib 3.10.8; the factorial range analysis used XGBoost 3.1.3. Stage-specific dependency files and execution records accompany the analysis code. The additional Ridge sensitivity analysis used Python 3.12.10, NumPy 2.3.5, pandas 2.2.3, SciPy 1.17.0, scikit-learn 1.8.0, joblib 1.5.3, and threadpoolctl 3.6.0.

**References**

Carstens, C. J. (2015). Proof of uniform sampling of binary matrices with fixed row sums and column sums for the fast Curveball algorithm. Physical Review E, 91, 042812. https://doi.org/10.1103/PhysRevE.91.042812

Strona, G., Nappo, D., Boccacci, F., Fattorini, S., & San-Miguel-Ayanz, J. (2014). A fast and unbiased procedure to randomize ecological binary matrices with fixed row and column totals. Nature Communications, 5, 4114. https://doi.org/10.1038/ncomms5114

Carstens, C. J. (2016). Erratum: Proof of uniform sampling of binary matrices with fixed row sums and column sums for the fast Curveball algorithm [Phys. Rev. E 91, 042812 (2015)]. Physical Review E, 94, 039902. https://doi.org/10.1103/PhysRevE.94.039902
